# Biomedical Applications of Bacteria-Derived Polymers

**DOI:** 10.3390/polym13071081

**Published:** 2021-03-29

**Authors:** Jonathan David Hinchliffe, Alakananda Parassini Madappura, Syed Mohammad Daniel Syed Mohamed, Ipsita Roy

**Affiliations:** Department of Materials Science and Engineering, Faculty of Engineering, University of Sheffield, Sheffield S1 3JD, UK; jhinchliffe3@sheffield.ac.uk (J.D.H.); pmalaku@gmail.com (A.P.M.); smdsyedmohamed1@sheffield.ac.uk (S.M.D.S.M.)

**Keywords:** bacteria, biopolymer, biosynthesis, biomaterial, regenerative medicine, tissue engineering, drug delivery, biodegradable polymers, polymer science, hydrogel

## Abstract

Plastics have found widespread use in the fields of cosmetic, engineering, and medical sciences due to their wide-ranging mechanical and physical properties, as well as suitability in biomedical applications. However, in the light of the environmental cost of further upscaling current methods of synthesizing many plastics, work has recently focused on the manufacture of these polymers using biological methods (often bacterial fermentation), which brings with them the advantages of both low temperature synthesis and a reduced reliance on potentially toxic and non-eco-friendly compounds. This can be seen as a boon in the biomaterials industry, where there is a need for highly bespoke, biocompatible, processable polymers with unique biological properties, for the regeneration and replacement of a large number of tissue types, following disease. However, barriers still remain to the mass-production of some of these polymers, necessitating new research. This review attempts a critical analysis of the contemporary literature concerning the use of a number of bacteria-derived polymers in the context of biomedical applications, including the biosynthetic pathways and organisms involved, as well as the challenges surrounding their mass production. This review will also consider the unique properties of these bacteria-derived polymers, contributing to bioactivity, including antibacterial properties, oxygen permittivity, and properties pertaining to cell adhesion, proliferation, and differentiation. Finally, the review will select notable examples in literature to indicate future directions, should the aforementioned barriers be addressed, as well as improvements to current bacterial fermentation methods that could help to address these barriers.

## 1. Introduction

Plastics have become incredibly important to our modern world. In 2019, it was estimated that globally, more than 350 million tons of plastic was generated in a year [1]. This success story is due in part to the incredible versatility of plastics, where the wide range of tuneable properties, generally reduced density, and variety of polymer classes have allowed for the replacement of metals and ceramics in all areas, from aerospace engineering to the biomedical arena [2,3].

Nowhere is this set of properties more useful than in the field of biomaterials. Plastics as a group contain valuable properties which make them ideal for use, both in medical devices and as in vivo implants for the treatment of pathological conditions. Early polymers used as biomaterials were hailed as being “bio-inert”, a property that allows the material to carry out its function without a widespread immune response and subsequent rejection [4]. Recently though, the onus has been on “bioactive” polymers, materials which actively interact with in vivo systems to bring about therapeutic change [5]. These may include measures to prevent bacterial adhesion or fouling, such as hydrophilic PEG coatings [6,7], immunoisolation polymers to protect therapeutic agents from the immune system [8], and biomodulatory polymers that may increase both cell differentiation and growth through chemical factor incorporation and advanced manufacturing techniques like electrospinning and additive manufacturing [9,10].

However, while purely synthetic polymer production has been incredibly successful in biomaterials science, there are of course notable disadvantages. Whilst the incredible longevity and durability of polymers has been a boon to multiple respective industries, the formation and subsequent concentration of microplastics in ecosystems worldwide has been a major concern of both conservationists and materials scientists alike [11]. Additionally, the mass-manufacture, usage, and disposal of commonly used polymers generates harmful emissions such as heavy metals, greenhouse gasses, and aerosolised microplastics [12,13,14]. Despite the development of international public awareness strategies to reduce polymer use globally, there is a clear need in the biomaterials sector for mass-production of polymers that retain or improve on current bioactive properties and reducing the environmental cost. One proposed solution is through the use of bacterial fermentation, a process by which naturally occurring or genetically engineered bacteria are used to produce polymers historically only available by synthetic pathways [15]. This technique holds various advantages over the previous chemical synthetic processes, including (generally) lower temperatures and pressures, enantiomeric selectivity and a wide manufacturing variety of biodegradable polymers, many of which are degradable or bioresorbable in physiological conditions [16,17,18,19]. Furthermore, even though many polymers cannot be currently synthesised by bacteria, the relatively simple molecules such as lactic acid that often make up the feedstock allow for further integration of less energy-intensive manufacturing methods in the polymer supply chain [20,21,22]. Finally, some polymers (such as the biomedically significant polyhydroxyalkanoates) can only be produced by biochemical processes [23]. This review will discuss the state of the art of concepts surrounding the manufacture and use of multiple bacteria-derived polymers in biomedical applications.

## 2. The History, Contemporary Status, and Future Applications of Bacteria-Derived Polymers

In this section, the materials comprising the class of novel bacteria-derived polymers including their classification, properties (biological, physical and chemical), current production processes (including subsequent modification), and current research in a biomedical context will be reviewed. Contemporary research will be highlighted to suggest future work, including methods to limit undesirable properties and exploring their potential for in vivo and clinical use.

### 2.1. Polysaccharides

Given that polysaccharides make up a large portion of bacterial synthesis, it follows that a large number of this group of molecules can be derived from bacteria. This section explores polysaccharides, molecules which in their structure include extensive glycosidic linkages of constituent sugar units. They present as products of metabolic processes for any organism, and can also be commonly derived from bacterial fermentation [24].

#### 2.1.1. Dextran

**Figure 1:**
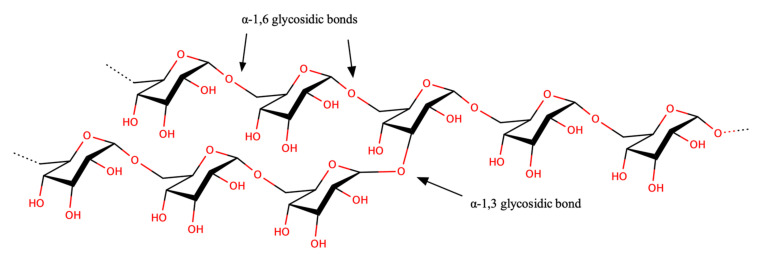
Molecular structure of dextran.

Consisting of α-d-glucopyranose subunits (Figure 1). Dextran is an exopolysaccharide with mostly α-1,6 glycosidic bonds (though smaller numbers of branching α-1,3, α-1,2 and α-1,4 bonds are present) [25,26]. Dextran utilization is widely recognized in food [27,28,29], cosmetic [26], and medicine [25]. Louis Pasteur first discovered dextran in 1861 from a viscous fermentation of wine [30], and later identified it via the chemical analysis of the product responsible for sucrose sugar syrup gelation [31]. The dextrorotatory nature of dextran inspired the nomenclature [32]. Several extensive dextran characterization studies carried out on dextran produced by *Leuconostoc pseudomesenteroides* XG5 [33], *Leuconostoc mesenteroides* AA1 [34], and *Leuconostoc citreum* B2 [35] confirmed its high-water retention capacity, which can allow it to act as a thickening agent, and potentially as a hydrocolloid and stabilizer agent [33].

#### Dextran Synthesis and Properties

Dextran is soluble in water, methyl sulphoxide, formamide, ethylene glycol, and glycerol [36], and barely reactive in mild acidic or basic environments [37]. The degree of branching in dextran depends on the lactic acid bacterial strain, with greater linearity indicating improved solubility in water [35]. For instance, dextran produced by *L. citreum* has 75% linear polysaccharide conformation [35], compared to the 95% of *L. mesenteroides* [38]; the latter therefore exhibits higher water solubility. On the other hand, the availability of a large number of hydroxyl groups within the glucose subunits opens up opportunities to consider dextran as a tailorable material in creating a number of desirable functionalisation [37,39].

Dextran was the first commercialized exopolysaccharide that The Food and Drug Administration (FDA) considered as “Generally Regarded as Safe (GRAS)”, which requires no labelling when incorporated in food products [28]. The mechanisms of its production are also known. The commercial dextran-producing bacterium *L. mesenteroides* does so by secreting dextransucrase enzymes that hydrolyse sucrose in the dextran cellular synthesis [28,40,41]. This however, is variable, with the molecular weight of the resulting dextran being influenced by the strain of the microbial producer [34]. Besides *Leuconostoc*, an array of other bacterial genera including *Weisella* [42,43], *Pediococcus* [44], and *Lactobacillus* [45] can produce dextran [46]. The extracellular glucosyltransferase enzyme catalyses transfer of D-glucopyranosyl residues from sucrose to dextran, resulting in the production of fructose as a by-product [32,43]. The glucosyltransferase enzyme production is mainly induced by the presence of sucrose in the media, instead of constitutive production (except for the *Streptococcus* species) [47,48]. The feedstock used for dextran production can include a wide array of sustainable elements, with some studies utilising sugarcane waste, raw sucrose, and sugarcane molasses to increase dextran production two-fold [49].

#### Dextran as a Potential Biomaterial

Despite being widely recognized and produced mainly within the food industry, dextran is also known for its suitability for biomedical applications, due to its relative biocompatibility and biodegradability. Incorporation of dextran in drug delivery systems takes advantage of its structural integrity in forming hydrogels. Pescosolido et al. [50] developed dextran-hydroxyethyl methacrylate with an alginate-based hydrogel system, which was found to have suitable flow properties that assisted injection for drug intake, and preserved dextran’s highly tuneable degradation rates (15–180 days). Pacelli et al. [51] used dextran-polyethene glycol cryogels to produce scaffolds exhibiting cytocompatibility with controlled vitamin B12 release profiles. Additionally, dextran-drug conjugates such as dextran-flurbiprofen and dextran-suprofen resulted in better therapeutic effects by enhancing their analgesic and antipyretic properties whilst reducing their constituent drug’s ulcerogenic effect [52]. Valproic acid-dextran conjugates have been shown to possess anticonvulsant properties as well as reducing the hepatocyte-toxicity and ulcerogenic effect of the epilepsy drug [53]. In another work of dextran-based functionalised material by Cai et al. [54], dextran was grafted with poly-ε-caprolactone in a novel hydrogel, aiming for enhanced mechanical properties with promising degradability in tissue engineering applications.

Dextran is a promising potential drug carrier material and has been found to have many advantages in targeting therapeutic approaches for several organs [37]. This degradability is attractive in colon-centered therapies, given the availability of dextranase-secreting saccharolytic *bacteroides* microflora within the intestine [55]. In this scenario, dextran is paired with therapeutic agents (where oral intake was previously impossible) synthetic hydrocortisone [56] and insulin [57]. The drugs are then released within the alimentary tract via gut bacteria-mediated enzymatic hydrolysis. Approaches in liver-targeting drugs have also been successful with dextran-based nanoparticles displaying low toxicity and multifunctionality (most notably in carrying nucleic acids) [58]. These approaches enable further endeavors towards the development of an enhanced targeted therapeutic vehicle by taking advantage of the chemistry of dextran towards human physiological responses. Other applications of dextran include their use as an antithrombotic agent in blood, as a volume expander and viscosity reducer for combating anaemia, as well as a haemodiluent for blood rheological rectification [59]. Based on the available literature, dextran is deemed to have a number of potential roles in the advanced biomedical field, mainly in drug delivery application in a number of target areas, whilst careful conjugation methods enable the development of a variety of dextran-based drug delivery materials.

#### 2.1.2. Glycogen

##### Glycogen Properties and Current Research

**Figure 2:**
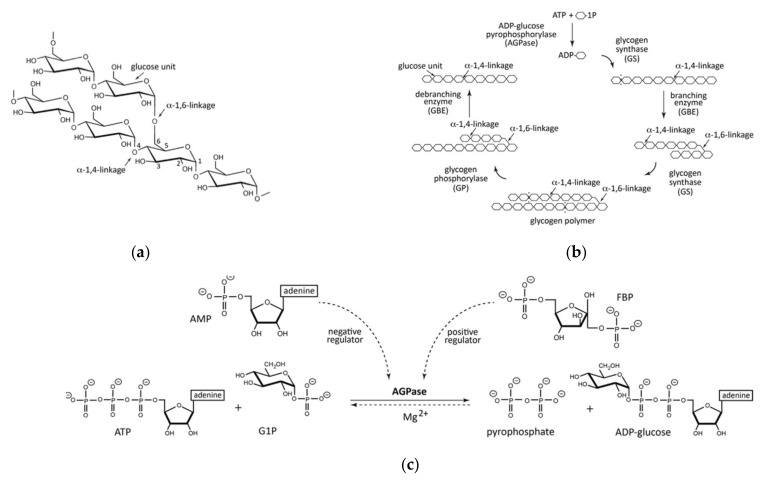
(**a**) The structure of glycogen, (**b**) biosynthetic pathway, and (**c**) regulatory pathway for glycogen accumulation in bacterial systems. Used with permission from Cifuente et al. [68]. © 2021 The Author(s). Published by Elsevier B.V.

Glycogen is a homopolysaccharide, made of multiple chains of glucose molecules, held together by both α-1,4 glycosidic bonds, and α-1,6 glycosidic bonds, providing linear and branched components that allows for efficient packing [60]. In this regard, glycogen is similar to plant-derived starch and cellulose, with the exceptions of its source, aggregate geometry, and linkage type [61,62]. Nevertheless, glycogen is vital in mammals, acting as an energy store and a homeostatic tool for the regulation of blood sugar concentrations in multiple tissue types (Figure 2) [63,64]. Although bacterial cellulose has been widely explored as a bacteria-derived polymer, glycogen’s potential therapeutic roles as a bulk material remain relatively unexplored [65,66,67].

The structure of glycogen comprises of branched chains of glucose molecules. Given its shared energy storage role between multiple kingdoms, glycogen is unsurprisingly found within the human body [69]. Moreover, its presence in healthy tissue assumes the body is able to break glycogen down. Under the homeostatic action of glucagon (produced by the islets of Langerhans), glycogen undergoes several enzymatic steps involving the breakdown of the α-1,4 and α-1,6 glycosidic bonds comprising glycogen’s microstructure [70,71,72]. Conversely, whilst this process occurs as part of healthy haemostatic function, implanted glycogen has been shown to induce some immune effects in vivo; indeed, glycogen injection has long been used to induce activation of polymorphonuclear neutrophils, possibly due to perceived liver hepatocyte damage [73,74]. However, given that glycogen degradation is regulated by homeostatic metabolic function in the human body, it is possible that small, if not bulk quantities of implantable material may be accepted in vivo.

**Figure 3:**
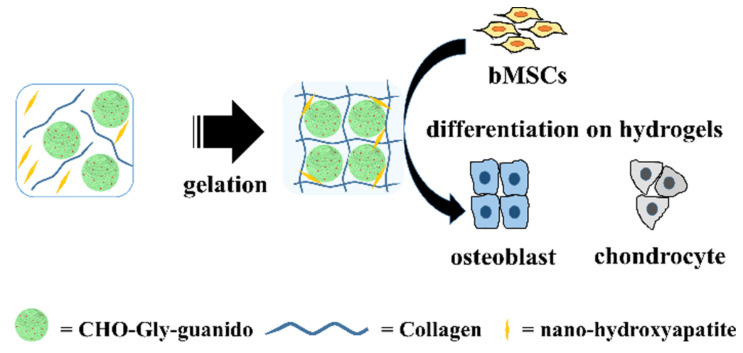
The process of using the flexible crosslinking nature of glycogen to produce a nanohydroxyapatite/collagen scaffold for the differentiation of bone and cartilage tissue. Reprinted with permission from Zhang et al. [83]. Copyright © American Chemical Society.

In addition to non-toxic breakdown products, glycogen possesses excellent chemical properties for use as a biomaterial. As mentioned previously, glycogen possesses a “hyperbranched structure”, capable of packing a large quantity of glucose entities in a relatively small space. However, the branching crosslinks resemble polymers used in the manufacture of hydrogels, water-swollen, highly crosslinked polymer networks, common in the biomaterial and tissue engineering research [75,76,77]. Work has already been undertaken using this approach; Patra et al. [78] successfully crosslinked glycogen and *N*-isopropylacrylamide with the linker molecule EGDMA, to produce a stable hydrogel, capable of both supporting and significantly accelerating mesenchymal stem cell proliferation. [79] followed up this work in 2020, showing that crosslink modification of a biopolymer containing glycogen and glycine changed both its swelling characteristics and mechanical properties. This has far ranging impacts for the usefulness of glycogen-based biomaterials for tissue engineering, especially considering that the substrate mechanical properties often determine proliferation depending on cell types, response to external chemical factors and even determining cell differentiation and phenotype [80,81,82]. Some studies have suggested the use of glycogen as a cross-linking agent. Zhang et al. [83] postulated that the branched structure could allow for multiple functional-molecule binding sites in three dimensions, testing this hypothesis through the generation of collagen-hydroxyapatite hydrogels, improving mesenchymal stem cell-osteoblast differentiation. This was achieved by decorating glycogen with guanido (a functional molecule found on the side chain of arginine) oxidising the resultant molecule to produce microspheres of CHO-Gly-guanido (Figure 3). Although glycogen in both bulk and hydrogel form has not been used extensively for clinical applications, hydrogels have often been touted as a candidate for improved drug delivery applications, with multiple clinical trials already underway [84,85,86]. Research has confirmed glycogen’s usefulness as a potential drug delivery device. Indeed, Patra et al. [78] demonstrated both 97% 2-month stability and controlled release capacity of loaded ornidazole (an antibiotic) from glycogen hydrogels. Moreover, Han et al. [87] decorated glycogen nanostructures with β-galactose, allowing, through Asiologlycoprotein (ASGPR)-galactose binding, to target liver cancer cells with limited uptake from other organs in a mouse model. Whilst this does not prove efficacy in humans, it is certainly an important step in demonstrating effective drug delivery systems using bacteria-derived polymers.

Despite its relative biocompatibility there is little literature describing the chemical and physical properties of glycogen as a functional material. This may be due to its relatively poor tensile strength. At a tensile strength of 0.128 MPa, Hussain et al. [88] found their glycogen-derived hydrogel to have an average value amongst the group, and certainly inferior to other biopolymer-derived hydrogels like chitosan and gelatine, as collated in a review by Hua et al. [88,89,90,91]. However, the glycogen-derived hydrogel demonstrated superior elongation at fracture, reaching 810% strain. Moreover, Hussain et al. [88] showed a strong correlation between the hydrogen bonding ability of each material (following cleavage and exposure of the functional –OH groups) and both elongation at fracture and self-healing efficiency, with the 1:1 glycogen/PVA hydrogel achieving 96% shape recovery, following cutting with a knife. This indicates that the addition of glycogen to existing hydrogels may confer elongation properties for tissues under continuous flexion (heart, muscle, bone etc.) (Figure 3), whilst allowing for a self-healing capacity following damage that may reduce follow up procedures following implant failure [92,93,94].

#### Concepts, Advantages and Limitations of Glycogen Production by Bacterial Fermentation

While the above properties of glycogen are excellent, most of the polymer obtained for research has been derived from enzymatic or synthetic laboratory manufacturing pathways. However, bacteria-derived glycogen is known, with nitrogen, carbon, salt, phosphate, sulphur and H^+^ ions, all contributing to glycogen biosynthesis in prokaryotic organisms [95,96,97,98], often during the stationary phase of growth [99]. These conditions are tabulated in a review by Preiss [99], who further tabulates a number of bacteria from which glycogen accumulation has been documented, including multiple strains of the genera *Streptomyces*, *Rhizobium* and *Methanococcus*, as well as multiple strains of the common opportunistic pathogens of genera *Streptococcus*, *Enterobacter* and *Escherichia* [100,101,102,103,104,105]. Since then, multiple other genera have been documented to produce glycogen, including *Synechococcus*, *Micropruina,* and *Candidatus* [106,107,108]. Given the number of genera exhibiting a potential for glycogen accumulation, the metabolic pathways involved in this process is fairly common to all. Indeed, in their paper, Preiss and Romeo [109] note that most glycogen accumulating bacteria known at time of publication operate using a highly conserved set of enzymes, including an ADP-glucose phosphorylase, glycogen synthase, and glycogen branching enzymes.

Despite the large number of described strains exhibiting the same biosynthetic, the literature on industrial-scale bacterial glycogen synthesis is remarkably sparse, which may explain why its applications have yet to be as widely reported as compared to other bacteria-derived polymers, such as the Polyhydroxyalkanoates. Nevertheless, it has been attempted. In efforts to manufacture feedstock for biofuel production, Aikawa et al. [110] cultured a euryhaline cyanobacteria (*Synechococcus* strain PC7002) for seven days, producing a maximum 3.5 g of glycogen from 500 mL of their “optimally conditioned” media. Although a step in the right direction, inefficiencies were noted, due in part to the fact that glycogen accumulates intracellularly, necessitating the disruption and subsequent lysis of the cell, compared to other extracellularly secreted polymers like gamma-PGA, which may be extracted with techniques tailored to minimize cell disruption such as centrifugation [111,112]. Given Aikawa et al. [110] lyophilized their microorganisms after seven days, an alternative would be selection of strains that secrete extracellular glycogen. While it is known that multiple *Pseudomonas* species produce copious quantities of polysaccharide biofilm under quorum sensing conditions, Sambou et al. [113] first detected “glycogen like capsules” secreted from *Mycobacterium* tuberculosis isolates. [114] produced the first evidence of non-pathogen-derived extracellular glycogen secretion in *Pseudomonas fluorescens* isolates [113,114,115]. Whilst these results may provide avenues of research for extracellular bacteria-derived glycogen extraction, more research may be needed to confirm other species of extracellular glycogen accumulators, as well as determining the genes and mechanisms responsible for extracellular glycogen secretion, in order to fully achieve industrial bacterial glycogen manufacture.

#### 2.1.3. Alginate

Alginates are natural unbranched exopolysaccharides, obtained mainly from seaweeds and bacteria, with the major source of prokaryote-derived alginate coming from the genera *Pseudomonas* and *Azotobacter*. Alginic salt can also be derived from this compound and is given the general term, algin [116]. It was discovered by E.C.C. Stanford in 1883 while working on dietary needs improvement methods [117]. Stanford was able to precipitate out a mucus-like substance called algin using sodium carbonate with further acidification from kelp [15]. The mucilaginous algin displayed both colloidal and gelation properties, showing a high level of viscosity on the addition of salts like sodium and potassium [118]. Krefting received a patent over algin purification in 1896, before its recognition and GRAS (Generally Recognized As Safe) classification by the FDA [119].

#### Structure, Biosynthesis, and Modifications

**Figure 4:**
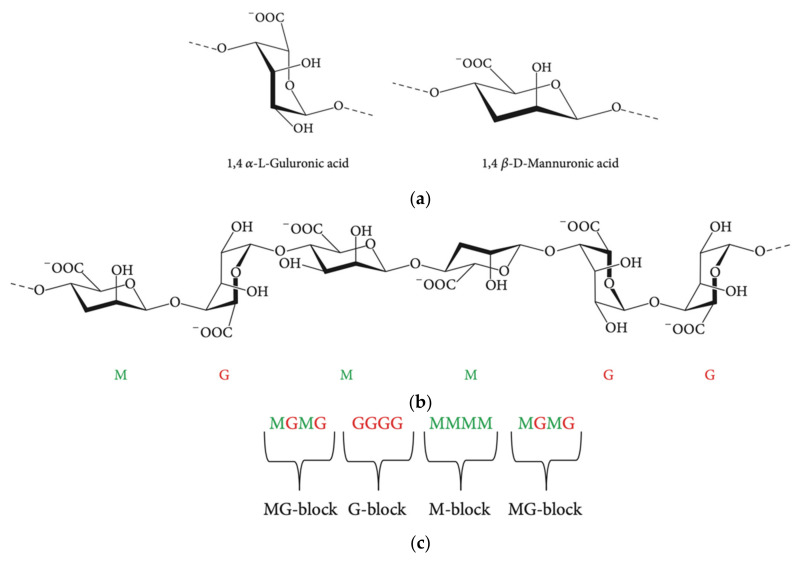
Structure of alginate (**a**) monomer, (**b**) chain conformation, and (**c**) distribution [120].

The basic structure of alginate consists of β-d-mannuronic acid and the C5 epimer α-l-guluronic acid. These two uronic acids are linked by 1,4-glycosidic bonds (Figure 4). Alginate has been produced in both hetero and homopolymer configurations, with the former being the naturally occurring form (the latter can still be produced from the early stage of polymerization by manipulating the gene expression of the bacteria to inactivate the catalytic state of the epimerase enzyme).

**Figure 5:**
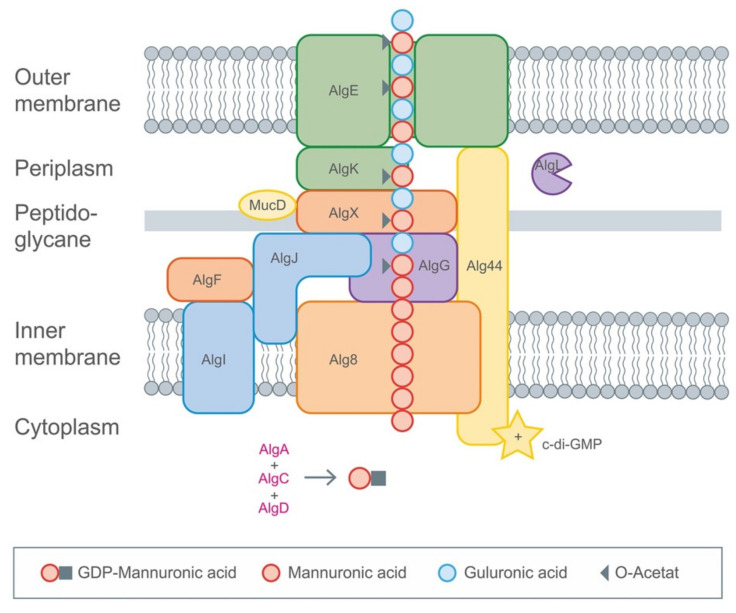
Schematic representation of biosynthesis of alginate in *P. aeruginosa*. Modified with permission from Schmid et al. [121].

The biosynthetic pathway, as it is best understood in *Pseudomonas aeruginosa*, is encoded by a single operon with 12 genes [116], starting with the synthesis of the active precursor guanosine-diphosphate (GDP)-mannuronic acid in cytosol. This is followed by polymerization by Alg8 polymerase-mediated transfer of sugar molecules from the donor to the growing acceptor molecule chain with the Alg8 polymerase (Figure 5). Finally, the periplasmic proteins help in modification and the product is exported [118].

As with any material of natural origin, the composition of the different components present in the polymer can vary significantly, from number to length of the monomer units. It is this composition that determines the physical and chemical properties of alginates. The factors that influence the mass and chain length of the material are the source, growing medium, and polymerisation conditions provided. Alginates extracted from seaweed have a high guluronic acid content compared to that produced from *P. aeruginosa*. Additionally, the intrinsic viscoelasticity of alginate depends on the decreasing flexibility of the constituents (guluronic acid-mannuronic acid > mannuronic acid-mannuronic acid > guluronic acid-guluronic acid) of alginate [122,123].

To form crosslinks, alginates can bind with divalent cations depending on the affinity of the ions allowing them to form a stable hydrogel or scaffolds [124]. The efficiency of the crosslinks is based on the selectivity, interaction, and affinity of the divalent cations with alginates, from lowest (Mg^2+^) to highest (Pb^2+^), giving the resulting product mechanical properties resembling stiffer tissues. The relative ratio of the constituents and their cations also play an important role in determining the physical and biological properties of the hydrogel. Such unique material properties have led to its application in agriculture, food, textile, cosmetic, and pharmaceutical/biomedical industries [116].

In order to enhance alginate’s properties, improvements and modifications have been made to the molecule. Numerous methods for chemical and physical modifications (ionic, covalent crosslinking, free radical reaction) have been developed to enhance their bioactivity and physical properties [125]. One such modification is the alteration of the two components through enzymes. Campa et al. [126] focused on isolating and recombining mannuronan C-5 epimerases expressed in wild-type *Azotobacter vinelandii* into *Escherichia coli* for enzymatic epimerisation that converts mannuronic acid residues into guluronic acid. Other enzymatic modifications include depolymerisation processes to isolate oligosaccharides from the alginate backbone. This can also be done by acid hydrolysis [127]. Additionally, acetylation, copolymerization reactions, and oxidation are employed to perform chemical modifications on hydroxyl groups among many others, while esterification and amidation modify the carboxylic groups [128]. On covalently attaching alkyl or aromatic groups to the backbone, solubility parameters can be altered, which will further affect resorption in the physiological system. As a result, a lot of research is being carried out to produce alginate derivatives, recognising their potential, especially in biomedical applications [129,130].

#### Potential Applications of Alginate in Biomedicine

The progress in the synthesis, processing, and modification of alginate has opened doors in biomedicine. Alginates are typically used in drug, protein and other bioactive molecule delivery systems as the release profile can be regulated to a very fast release or a prolonged one due to their porosity and gel formulation [131]. Multiple drugs with different release patterns were observed with alginates, non-interactive methotrexate diffused swiftly while a covalently attached doxorubicin only released after chemical hydrolysis [132]. Alginate in combination with chitosan has been explored widely, mainly because of its unique swelling behaviour. Few examples are in colonic and gastric drug delivery, where a sustained release and exceptional swelling degree was observed [133]. Divalent calcium ion modified alginate hydrogel as a carrier against *Helicobacter pylori* infection allowed for specific interaction and release in the site of infection [134]. Reports suggest that encapsulated proteins like lysosomes in ionically crosslinked alginate spheres can link to the matrix physically, which helps in a more sustained release. Alginate gel and their control over the release of angiogenic molecules have gained much attention due to their spatiotemporal control in delivery that aids in neovascularisation [135].

Since the properties of alginate facilitate appropriate wound moisture retention and wound healing, they are excellent candidates in dressing applications. There are a variety of commercially available alginate dressing like Algicell™, AlgiSite M™, Comfeel Plus™, Kaltostat™^,^ Sorbsan™, and Tegagen™ [136]. Rabbany et al. [136] used Stromal Cell-Derived factor 1 (SDF-1) to induce accelerated recovery of the epithelial wound in rat and pig models. The cell-adhesive and degradation behaviour are the main features that allow alginate to be used in a wide range of tissue engineering applications. Alginate has been used successfully as a minimally invasive material in bone tissue engineering to deliver cells, osteoinductive factors, and other molecules like bone morphogenic proteins [137]. In addition, alginate with calcium sulphate pre-shaped 3D cartilage had an elastic modulus almost similar to that of native cartilage and retained shape up to 30 weeks [138]. In liver tissue engineering, alginate showed efficient seeding capacity of hepatocytes while maintaining functional viability because of their porous, interconnected, and hydrophilic nature [139]. Alginate gels could regenerate axons from a transected nerve stump restoring the nerve gap with no major inflammatory responses [140]. The track record of the material suggests that alginate has the potential and utility for a number of wide-ranging biomedical applications for a number of different tissue types.

#### 2.1.4. Hyaluronic Acid

**Figure 6:**
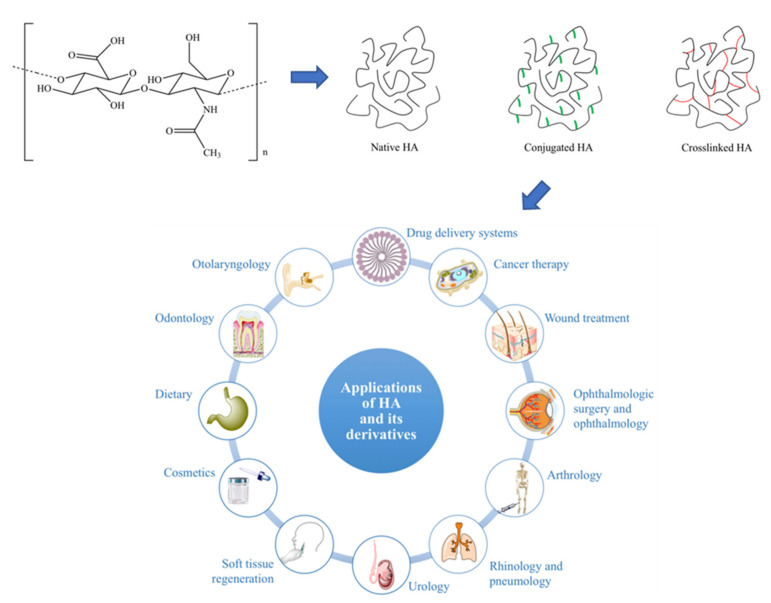
The popular modifications and potential biomedical applications of hyaluronic acid. Used with permission from Fallacara et al. [142]. Copyright © 2021 by the authors. Licensee MDPI, Basel, Switzerland.

Unlike glycogen, where endogenous granules of substance are not found within the human body and alginate, which is not naturally produced nor hosted by the human body, hyaluronic acid (HA) naturally occurs in mammals, having first been isolated from the “vitreous humour” of the bovine eye by Meyer and Palmer [141] in 1934. Since then, sustained analysis of the polymer has revealed unusual chemical and physical properties, making HA both an easily modifiable biomaterial for multiple clinical roles (Figure 6) and a well-known polymer produced using bacterial fermentation.

#### Properties, Current, and Future Clinical Usage

**Figure 7:**
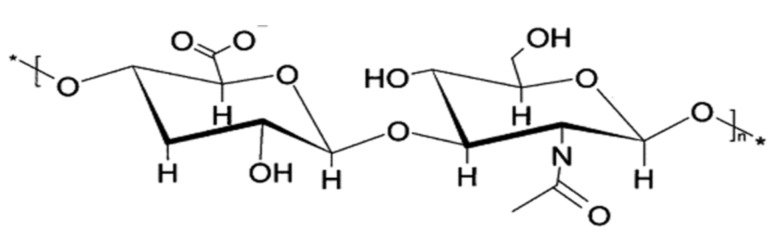
The backbone of the hyaluronan molecule, with the main constituents of d-glucuronic acid (**left**) connected via ester linkage to *N*-acetyl glucosamine (**right**). Reused with permission from Ward et al. [145]. Copyright © 2021 Elsevier Science B.V. All rights reserved.

Despite being a polysaccharide, HA does have some major structural differences to glycogen. HA is unbranched, its microstructure consisting of long parallel single chains of disaccharide sugars, which are themselves made up of glucuronic acid and *N*-acetyl-d-glucosamine [143,144]. This arrangement of *N*-acetyl hexosamine and hexose-based disaccharides defines HA as a glycosaminoglycan (GAG), a group of molecules which make up a gel-like “ground substance”, resulting in the extracellular space (Figure 7).

These constituents are partially formed from a proteoglycan core wherein chains of GAGs extend, the presence of sulphonated groups that (together with the carboxylic acid groups of *N*-acetyl hexosamine and hexose) attract water molecules, allowing the final hydrated macrostructure a degree of rigidity [146,147,148,149]. Despite HA’s fairly unique position amongst the GAGs of being the only member of the group not to contain sulphate groups, HA retains its negative carboxylic acid groups, and therefore some water retentive ability [143,144]. HA is also exceptionally large with molecular weight between 10^5^ and 10^6^ Dalton and is between two to four orders of magnitude heavier than the GAGs chondroitin sulphate or heparin [150,151,152]. This allows not only for more rigidity from HA-HA interactions, but also limits the flow of water and solutes out of the structure. Finally, HA has the ability to scavenge (ROS), potentially damaging radicals released via photolysis and as a biological defence mechanism against foreign material. Jahn et al. [153] noticed that this occurs mostly on glucuronic acid residues, forming (amongst others) gluconic and glyceryl acids. Thus, HA acts as sacrificial protection material in vivo, since it loses both structure and therefore function as a result of ROS attack [153].

These chemical properties have wide ranging impacts for the physical and therefore biological properties of HA, dictating polymer function in both the in vivo environment and in clinical applications. HA’s water retentive ability confers compressive strength to tissues, acting as shock absorbers, such as in cartilage [154]. Greene et al. [155] demonstrated this by compressing HA-containing collagen samples under HA digestion conditions, finding that digesting HA tended to stiffen their construct, recovering much less readily due as its degraded water attraction potential prevents re-swelling [151]. Furthermore, HA-containing hydrogel preparations encountered greater swelling rates, following compression, with increasing HA concentration (though the reduction in compressive strength with increasing HA concentration would indicate HA’s role in elastic recovery, rather than resistance to compressive stress) [156,157,158]. Clinicians use HA’s hygroscopicity in multiple clinical roles, including as expanding fillers for plastic surgery [159]. This is possible in humans because post-translational modification of hyaluronic acid is limited between species, allowing for HA transplantation from bovine, bacterial, and poultry sources with incredibly limited immune response (mostly due to incomplete purification) [160,161]. Degradation is controlled over seven to nine months, whilst degradation products have been shown to be both non-toxic and can be metabolised with ease. Romagnoli and Belmontesi [162] list a number of HA products currently used within the filler market, including the Allergan system, Qmed, and FDP.

However, the current medical applications of HA are not limited to plastic surgery, with multiple clinical trials confirming HA’s ability to improve lubrication and joint articulation in vivo, compared to more modern methods. Raeissadat et al. [163] decreased Western Ontario and McMaster Universities Arthritis Index (WOMAC) score by 11.4%, following HA injections into the osteoarthritic knee, noting no significant difference between HA and ozone treatment after six months. HA also significantly reduced the Visual Analogue Pain Scale (VAS) score and increased American Orthopaedic Foot and Ankle Society (AOFAS) score of patients suffering from ankle injury after 15.3 months (though it is important to note that this study recommended the use of platelet-rich plasma injections over HA due to its higher efficacy) [163]. In vivo articulation is also aided by HA’s lubricative ability. Lin et al. [164] resolved the question of HA’s lubrication mechanism by immersing lipid layers into liquid HA solution to determine their frictional coefficients, finding that whilst HA was a relatively poor lubricant, its ability to complex with a large number of molecules allowed for complex formation with frictionally superior phosphatidylcholine also found in cartilage, allowing for a synergistic increase in lubricity. This confirmed work undertaken in indicating a synergistic partnership between the mucinous glycoprotein lubricin and HA for the reduction of arthritic potential in mouse models [165]. Whilst these studies paint the lubricative properties of HA in a negative light, the presence of both complex-forming molecules in humans could allow for in vivo complex formation if either was implanted for therapeutic purposes, producing better combination treatments instead of the dichotomy of the previously mentioned studies.

The final avenue of research this review will discuss is HA’s protective capacity against both immune cells and inflammatory factor release. Harrington et al. [166] methacrylated HA to produce microencapsulated islet microspheres, which were able to induce normoglycemia for four to six weeks without immune response in induced-diabetic mice, though its polyethene glycol diacrylate (PEGDA) counterpart was able to produce similar results non-transiently (study time was 16 weeks), perhaps due to the swelling following implantation generating a larger barrier to oxygen diffusion. However, modification using collagen HA blends crosslinked with PEGDA by [167] allowed microencapsulation islets to survive for up to 80 weeks with little to no fibrosis, though the study did not mention if their blend caused more or less swelling with collagen addition. Whilst this is the case, further modification may allow HA to usurp alginate as the current go-to microencapsulation matrix. Finally, the ease of HA functionalisation has allowed scientists to find a use in cancer therapies. Resnick et al. [168] found that a common HA receptor (CD44) was overexpressed in a number of cancers, though a link had already been noticed by Yang et al. [169], who also determined the selectivity of over-expressed hyaluronan-mediated motility receptor (RHAMM) in cancer [168,170]. This information has been expanded upon in multiple studies combining HA’s efficacious drug loading capability and its affinity for cancer cells to improved targeted drug delivery products (though the closest these treatments are to being tested in humans has been in xenografted human tumour tissue) [171,172,173,174]. Nevertheless, the literature certainly promotes HA as a contemporary and potential clinical solution to treat multiple pathology types, including tissue degeneration, cancer, and autoimmune disorders.

#### Past, Current, and Future Manufacturing of Hyaluronic Acid

Given the identical molecule manufacture capacity of multiple organisms representing three kingdoms, HA (as previously mentioned) has historically been isolated from a number of organisms, including bovine eye, rooster wattle, or human umbilical tissue [160,161]. Although these sources have generally been successful, renewed scrutiny due to zoonotic infection and incomplete purification methods have led to renewed interest in bacterial fermentation as a route for the manufacturing of HA [175,176]. A benefit during immunoisolation is that bacterial HA as a virulence factor generates a physical barrier to attacking immune cells and the complement system, while reducing the harmful effects of cytotoxic factors, antibiotics, and ROS, reducing the immune system’s ability to mount an effective response [177,178,179]. Gunasekaran et al. [180] mentions the use of the capsular HA releasing system in strains of both *Streptococcus* and *Pasteurella* species, though the first commercial production of HA was conducted using isolates of *Streptococcus zooepidemicus* [180,181]. Recombination of HA synthases allowed for a reduction in streptococci-derived endotoxins resulting from fermentations, allowing for reduced immune responses in vivo. [182] modulated the concentrations of dissolved oxygen and *N*-acetyl glucosamine during the fermentation process; doing so allowed them to modify the molecular weight of the HA produced. The industrial manufacturing costs of HA may be significantly reduced if a proposal by Arslan and Aydogan [183] gains popularity: their team replaced the traditionally expensive peptone and *N*-acetyl glucosamine feedstock with sheep wool-derived peptones and molasses, finding that the wool peptones generated better yields compared to commercial peptones [182,183,184,185]. Li et al. [186] was able to use the temperature of the reaction vessel to control the molecular weight of their HA. It is this ability to have fine control over not only the polymer produced but also the microstructure that will allow scientists to manufacture and use HA in a biomaterial context, to tune the process to the required mechanical properties of the desired application. Researchers will hence be able to fully realise the true potential of hyaluronic acid in the fields of wound healing, tissue engineering, and cancer research.

#### 2.1.5. Gellan

##### Structure, Composition, and Classification of Gellan Gum

**Figure 8:**
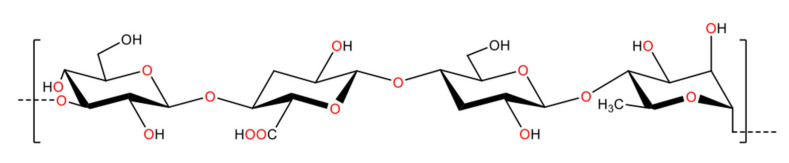
The chemical structure of n repeating units in gellan gum. Reused with permission from Zhang et al. [190]. Copyright © 2021 Elsevier. All rights reserved.

Gellan gum is a high molecular weight linear extracellular polysaccharide, accumulating in multiple strains, including *Sphingomonas elodea*, *Sphingomonas paucimobilis,* and *Pseudomonas elodea* [187]. Approved by the USA FDA in 1992 as a food additive, gellan is mainly composed of a 1,3-β-d-glucose, 1,4-β-d-glucuronic acid, 1,4-β-d glucose, 1,4-α-l-rhamnose backbone in a 3:1:1 (general) relationship, respectively (Figure 8). Acetyl group concentration defines the three types of gellan gum [188,189]. Attached on the glucose residue adjacent to the glucuronic unit are the acyl groups acetate and glycerate, forming one among the three types of gellan gum.

During the industrial fermentation process, these additional residues or groups are removed through hot alkaline hydrolysis, yielding a linear simple chain polymer, deacetylated gellan gum [191]. This structure may transition from a highly coiled to a double helix structure on cooling. Even after such a transition, both acetylated and deacetylated gellan gum are capable of gelation. The deacetylation process results in physical and chemical changes to the material and gel formation, depending on the degree of deacetylation, making the polymer less flexible, transparent, and much more thermally stable, otherwise soft and elastomeric [192,193].

Similar to the gel-forming property of xanthan gum, the presence of a cation helps form a stable hydrogel as the gelation process of gellan is ionotropic. Gel formulation and their properties are highly influenced by factors like the number of cations used and their chemical structure. For example, during ionic crosslinking, divalent cations like calcium or magnesium show higher gelation efficiency than sodium or potassium monovalent cations. In the former case, the chemical bonding between the carboxylate group of glucuronic acid molecules and the divalent cations along with the screening effect caused by the electrostatic repulsion among the ionized carboxylic acid groups results in the gelation, while in the latter, there is only the screening effect across the gellan causing gelation. Moreover, gellan can be a self-supporting hydrogel even in the absence of ions with the mere inclusion of cell culture media [194,195]. Similar to the deacetylation process, clarified gellan gum is formed during fermentation, heating the broth to a temperature of 90–95 °C. On heating, the bacterial cells are killed and protein residues are removed after filtration with cartridge filters. This more viscous broth is precipitated by isopropyl alcohol to form the third type, clarified gellan gum. This again is available in two types: KELCOGEL^®^ as an industrial food product and a more refined and purified Gel-Gro gellan gum used in pharmaceutical and biomedical applications [187,196].

#### Biomedical Applications of Gellan

The use of gellan in biomedical applications requires mechanical integrity and stability. Certain features that limit its use include (i) the lack of mechanical strength since it gradually dissolves under physiological conditions; (ii) inability to envelop cells due to rough gelation conditions. Nevertheless, such drawbacks can be addressed by material modifications, possible due to the presence of hydroxyl and carboxyl groups in glucuronic acid. Moreover, many physical modifications have been employed and improvised for imparting better physicochemical and biological properties [197].

Such modification allows for a wider range of applications of gellan and their derivatives in pharmacy and medicine, especially in drug delivery, gene therapy, as protein carriers, tissue engineering, and regenerative medicine [198]. The major biomedical applications of gellan include nasal, ocular, gastric pharmaceutical delivery systems, and tissue engineering applications. Gellan is generally used for oral formulations, as gels or coatings of capsules that assist in the release of the ingredients like bioactive molecules or drugs with modified or sustained release profile. Floating gels are one of the main forms in which gellan has been used in drug delivery. In situ floating gels are one such form used in a variety of applications against gastric ulcers, peptic ulcers, rheumatic arthritis, inflammation, and allergic rhinitis. Gellan gum beads carrying glipizide was developed against diabetes as a hypoglycaemic agent. These beads were also used for the slow release of the β-blocker propranolol, for the treatment of hypertension. Gellan gum gels can also protect bioactive molecules from the low pH of the stomach [199,200].

Tissue engineering application of gellan is mainly owed to its biocompatibility, nontoxicity, easy processability, a structural similarity with glycosaminoglycans, and most importantly the similarity of their mechanical properties with common tissues. The material porosity, binding capacity, and ionic interaction with positively charged biomolecules and other moieties also make them an excellent material for tissue engineering [201]. Gellan can be fabricated into films, fibres, 3D structures, and lyophilized scaffolds, as well as bioprinted and modified with RGD peptide to form multi-layered scaffolds mimicking cortical tissue [202]. Moreover, modified gellan exhibits a wide range of mechanical properties, with some gellan-amyloid protein nanofiber scaffolds reporting specific strengths comparable to steel [203]. Improved differentiation of adipose stem cells was observed in gellan based sponge, which was fabricated through freeze-drying [204]. In addition, gellan and HA has been freeze-dried to be applied as a scaffold implant in skin regeneration and vascularization [205]. In cartilage repair, an injectable form of gellan blended with stem cell and growth factor was used for knee repair in an animal model. Hence, it can be concluded that gellan is a viable substrate for a wide variety of biomedical applications and further research is required to facilitate the utilization of this versatile material.

#### 2.1.6. Xanthan

**Figure 9:**
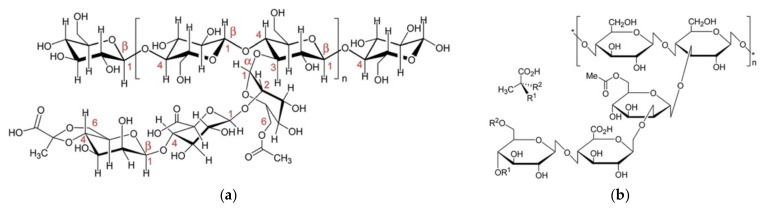
Chemical structure of xanthan gum, (**a**) chair, and (**b**) Haworth projection [209].

Produced by bacteria of genus *Xanthomonas*, xanthan gum is a microbial high molecular weight exopolysaccharide discovered by Allene Rosalind Jeanes in the 1950s. Xanthan is an extremely important commercial polysaccharide, used as a food thickener or stabilizer [206] and in industrial applications, where xanthan’s thermal stability and pseudoplastic behaviour make it a component of water-based drilling fluids. This material is nontoxic and was approved by the FDA as a safe polymer in 1969, to be used in food products (Fed Reg 345376). With a backbone of β-1,4-d-glucopyranose glucan repeating units, it is a branched polymer with β-1,4 d-mannose, β-1,2 d-glucuronic acid and d-mannose side chains (Figure 9). These trisaccharides are attached with α-1,3 linkages on each alternate glucose residue. While the mannose moiety in the terminal end is partially substituted with pyruvate residues linked to the 4- and 6-positions as an acetal in the side chain, the inner mannose unit undergoes acetylation at the C-6 position [207]. The charge density on the xanthan chain is increased when the deprotonation of *O*-acetyl and pyruvate residues take place at pH > 4.5, allowing physical crosslinking of the xanthan mediated by calcium ions. Xanthan has a polyanionic characteristic owing to the presence of glucuronic acid in the side chain [208].

#### Biosynthesis and Industrial Production

The synthesis process of xanthan gum is similar to exopolysaccharide synthesis by other Gram-negative bacteria, using activated carbohydrate donors for shaping the polymer on the acceptor molecule. The biosynthesis is initiated through the Entner–Doudoroff pathway transforming glucose to pyruvate [210]. Pyruvate then enters the tricarboxylic acid cycle to produce adenosine triphosphate (ATP) molecules. Other metabolic cycles follow, involving sugar donors (monosaccharides from nucleotide phosphor-sugars), sugar acceptors (polyprenol phosphate), acetyl-CoA, and phosphopyruvate, transferring sugar donors to the acceptors (lipid anchor) forming a sugar sequence [211]. The acetyl and pyruvyl residue enter the trisaccharide side chain and the latter influence the polymer viscosity (lesser the pyruvyl content, lower the viscosity). Industrial-grade xanthan is produced through fermentation followed by a pasteurization process to kill the microorganism, before precipitation in ethanol, spray drying, re-suspension in water, and re-precipitation [210]. Xanthan used for in vivo applications must progress through several enzymolysis and filtration processes to get an extremely pure version of the material [212]. In producing cell-free xanthan gum, the cell separation step is highly cost-intensive (though additions of alcohol and salt appear to promote precipitation) [213].

#### Biomedical Properties of Xanthan

With a high molecular weight of 1–20 × 10^6^ mol/g and intramolecular and intermolecular hydrogen bonding interactions due to the presence of the hydroxyl and carboxyl polar groups, xanthan exhibits a high intrinsic viscosity in an aqueous solution, even at low concentrations, behaving as a pseudoplastic fluid [209,213,214,215], explaining the use of xanthan in areas like food, cosmetics, and pharmaceuticals [216,217]. In a biomaterial context, improvement of xanthan’s existing properties such as solubility, swelling, gelation, or stability have (by hydroxy and carboxy group-mediated chemical modification) been considered. Additionally, the traditional drawbacks of xanthan including microbial contamination, uncontrolled hydration, low viscosity on storage, poor reactivity and thermal stability have been minimized through acetylation, esterification or etherification, oxidation, peptide linking, ionic and covalent crosslinking and other physical and mechanical modification [209]. At any concentration, xanthan fails to form a true gel due to weak, non-covalent intermolecular interactions [218]. However, hydrogel crosslinked 3D structures, produced using physical or chemical crosslinking, facilitates their use as a carrier of drugs or proteins in delivery systems [219]. Biocompatibility, non-toxicity, and softness of the material result in xanthan being suitable for this purpose [220].

One application of xanthan stems from its resistance to enzymatic digestion in the stomach or small intestine, providing a stabilising shield for an enclosed therapeutic factor and delivering them to the colon as they degrade in the presence of anaerobic microflora present in the colon [221]. *Bacteroides, Bifidobacteria*, and *Eubacteria* have been shown to degrade xanthan for energy, making the (non-dysbiotic) colon environment an excellent end-point for a xanthan-based drug delivery system [222]. In a study based on acrylic acid-crosslinked xanthan and starch hydrogel grafts, crosslinked with acrylic acid maximum swelling capacity (caused by the ionization of –COOH groups to form –COO^−^ ions) and prolonged release [223]. A xanthan nasal gel for drug delivery through the olfactory lobe helped improve drug permeation and bioavailability. The in-situ gel systems in ocular therapy resolve the difficulty in attaining optimal drug concentration, which is usually brought about by precorneal loss as an outcome of eye blinks and movements. Low molecular weight xanthan acts as an excellent anti-oxidant agent and protects against H_2_O_2_-injured Caco-2 cells, concurrently inhibiting oil peroxidation [224]. They also have an added benefit of immune protection against the neoplasm and resistance to overproduction of ROS, marking their importance as a potential anti-inflammatory agent. Other xanthan-based carrier systems have included mucoadhesive nicotine-carrying patches with superior fast initial release and a subsequent controlled release for 10 h compared to contemporary patches [225]. Xanthan with chitosan was coated on liposomes assisting active protein delivery and exhibited an excellent drug release profile and mucoadhesive property [226]. The in vitro release study of zolmitriptan from xanthan, PVA, and HPMC film showed around 43% rapid release in 15 min with no damage to the buccal mucosa [227]. The above examples clearly state its ability as a carrier of bioactive molecules and drugs mostly because of their stability, protection, and controlled release kinetics.

Xanthan gum blended with natural-based polymers or materials like nanohydroxyapatite has been fabricated and assessed for bone, cartilage, skin regeneration, other tissue engineering applications, and cellular studies specified to its biomimicking potential [228]. Due to the very obvious biocompatibility and biodegradation, xanthan is an interesting material with huge potential as a tissue engineering scaffold. A significant proliferation of fibroblast tissues was shown when xanthan was fabricated with electroactive polypyrrole compared to virgin xanthan [229,230]. Chitosan and xanthan scaffolds also showed fibroblast viability as dermal dressing. The Xanthan hybrid scaffold with hydroxyapatite assisted in the cell adhesion and growth of osteoblasts, while improving alkaline phosphatase activity [228]. Xanthan in the presence of magnetic nanoparticles helped in vitro neural differentiation of stem cells [231]. Though contamination, viscosity variations, and thermal/mechanical instability are some of the impediments in their large-scale applications, its potential benefits can be exploited to push these limits to make them useful in the food industry and biomedical applications.

#### 2.1.7. Curdlan

Curdlan is a high molecular weight extracellular polysaccharide composed of β-1,3 glucopyranosyl repeating units connected by glycosidic linkage [232]. Discovered in 1966, Harada et al. [233] extracted the homoglycan from *Alcaligenes faecalis* var. *myxogenes*, observing the ability of the material to ‘curdle’ when heated. The resultant “curdlan” product was insoluble in water, with a temperature-initiated gelling property that forms elastic gels in an aqueous solution [234]. Curdlan is approved by the FDA for its safe use as a food additive and is a common source of dietary fibre in Korea, Taiwan, and Japan [235]. Curdlan biosynthesis is initiated when uridine diphosphate (UDP) glucose is synthesized from a carbohydrate substrate. The transfer of the monosaccharide from the precursor to the carrier lipid takes place and polymer construction is carried out subsequently. The formed polymer is extruded after chain elongation [236]. Curdlan is extracted on a commercial scale through the fermentation of *Alcaligenes faecalis* var. *myxogenes*, now reclassified as *Agrobacterium* sp. The material is extracted and thoroughly purified before use [237].

#### Structure and Properties of Curdlan

**Figure 10:**
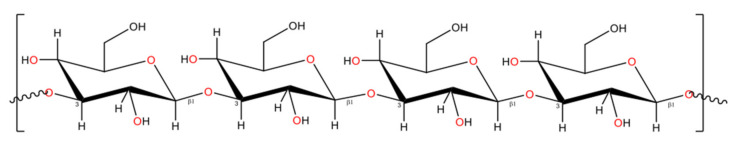
Structure of β-(1,3)-glucans, curdlan.

Curdlan comprises of β-(1,3)-glucans (Figure 10), a bacterial exopolysaccharide observed in both prokaryotes and eukaryotes. Curdlan can be considered significant among the β-(1,3)-glucans, due to their structural peculiarity, since they can be favourably manipulated. The solubility and rheological properties of curdlan therefore hold a special interest in biomedical material research [232].

Unlike cellulose and chitin, curdlan is insoluble in water. However, organic solubility is much more enhanced compared to other materials in the same group—solubility in alkaline media is also preserved. Curdlan gelling behaviour is relatively novel in that it either forms a thermal non-reversible gel at around 80 °C or a thermally reversible gel at approximately 55 °C [238]. This interesting property is on account of its structural transformation when heated from room temperature to higher degrees [239]. At room temperature, curdlan has a single helical structure or triple helix that is loosely intertwined, which takes a more condensed and rod-like helical structural form with increasing temperature [240]. Recent literature has shed light on the immunostimulatory properties of β-glucans, where they are used as a biological response modifier. Modified curdlan (aminated or sulphated) as biological cues can enhance or adapt immune responses against tumours or for wound repair. The anti-infective and anti-inflammatory activities of curdlan enhance its scope in material application [241]. With such chemical derivatization accompanied by generic gelling and rheological properties and their commercial availability, curdlan can be considered as a material that imparts new properties for food and biomedical application.

#### Biopharmaceutical Applications of Curdlan

Curdlan is known for retaining its activities even after forming derivatives, especially carboxymethyl curdlan, which is extensively used to retain antitumor efficacy. In a recent study on the anti-infection property of curdlan, results confirmed resistance to colonization on *E. coli* in the intestine. The structural peculiarity and pharmacological capability of curdlan have found extended use in drug delivery [242]. Curdlan was used as an encapsulation vehicle in a rectal suppository system, where the gel stayed intact for slow drug diffusion [243]. In addition, Na et al. [244] demonstrated carboxymethylated curdlan-sulphonylurea copolymer nanoparticles encapsulating all-trans-retinoic acid, and showed first-order release kinetics with no cytotoxicity. All-trans retinoic acid is an active metabolite of vitamin A under the family retinoid. They have significant promise for cancer therapy and chemoprevention.

Curdlan has also proved its importance in wound healing applications. Delatte et al. [245] improved healing speed, reduced pain, and lowered the number of dressing changes compared to standard treatments using a β-glucan collagen matrix, whilst curdlan blended with polyvinyl alcohol nanofiber scaffold crosslinked with glutaraldehyde vapour showed better wound closure data compared to the polyvinyl alcohol scaffold, probably due to the immunomodulatory properties of curdlan [246]. Despite sparse research on tissue engineering applications of curdlan, porous scaffolds developed with curdlan and polyvinyl alcohol foam have been reported, with results indicating favourable cell proliferation and differentiation in vivo, as well as preserving satisfactory enzymatic degradation [247].

Thus, curdlan shows great potential in biomedical applications. It has superior helical structural, pharmacological, and gelation properties and has not yet been explored to its full potential. The table summarizing polysaccharide production and biomedical applications can be found in Table 1.

### 2.2. Polyesters

#### 2.2.1. Polyhydroxyalkanoates

**Figure 11:**
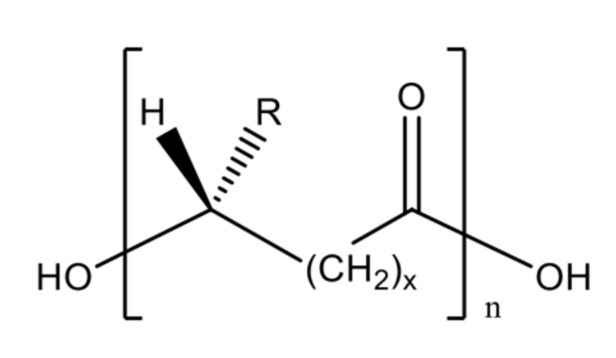
The general chemical structure of PHAs.

**Figure 12:**
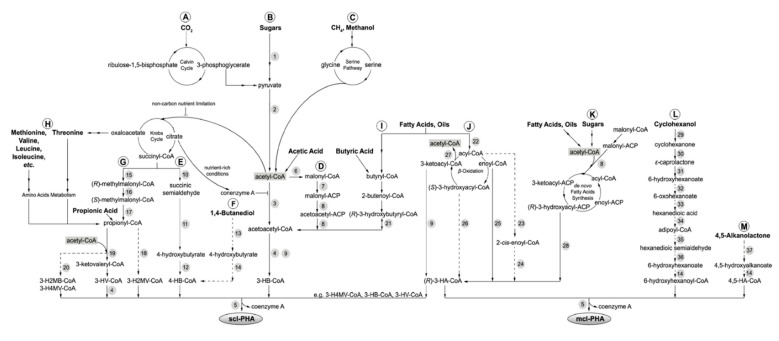
PHA biosynthetic pathways producing scl-PHAs and mcl-PHAs [251].

Polyhydroxyalkanoates or PHAs were discovered by French microbiologist Maurice Lemoigne in 1926. He extracted the biopolymer within a bacterium called *Bacillus megaterium* that contained a short-chain-length PHA, poly(3-hydroxybutyrate) or P(3HB) [248]. PHA is a polyester, a polymer with linear ester linkages and differs in terms of the side pendant chain. The side chain is typically a saturated aliphatic chain with a range of carbon count of up to 13 carbons [249]. There were also variations in terms of the position of the pendant chain, such as 4-, 5-, and 6-hydroxyalkanoates, resulting in different polymer characteristics (Figure 11 and Table 2). These different polymer molecular structures are derived from different bacterial strains and species, as well as different carbon sources used, including fatty acids and sugar (Figure 12) [250].

Besides the chain variation, PHAs are not limited to homopolymer synthesis. Poly(3-hydroxybutyrate-*co*-3-hydroxyvalerate) or P(3HB-*co*-3HV) or PHBV [252,253], poly(3-hydroxyhexanoate-*co*-3-hydroxyoctanoate) or P(3HHx-*co*-3HO) [254], poly(3-hydroxybutyrate-*co*-3-hydroxyhexanoate) or P(3HB-*co*-3HHx) [255,256] and poly(3-hydroxyoctanoate-*co*-3-hydroxydecanoate) or P(3HO-*co*-3HD) [257] are some examples of heteropolymeric PHAs, the distinctive structures of which are due to the low substrate specificity of the synthases, bacterial species, and carbon source utilised during the accumulation process [249]. Indeed, multiple types of bacteria have been utilised to obtain specific types of PHAs, such as *Pseudomonas* sp. [258] for mcl-PHA and *Bacillus* sp. [259] for scl-PHA production. *Pseudomonas* sp. has an added value in promoting sustainable PHA production since it is capable of feeding on readily available carbon substrates such as coconut oil [260], unprocessed biodiesel waste [261], and frying oil waste [262]. Certain microbes have been subjected to genetic modification in order to enable specific substrate uptake. For example, in an attempt to make the production of PHA cost-efficient, in *Pseudomonas putida* KT2440, the *XylA* and *XylB* genes have been introduced, which enabled xylose uptake as a sustainable alternative carbon source [263,264]. Another work modified *P. putida* KT2440 to overexpress PHA synthase genes promoting PHA accumulation, whilst deleting the depolymerase *phaZ* and β-oxidation genes to avoid PHA degradation [265].

#### PHA as a Biomaterial

PHA utilisation in biomedical research is extensive due to its biocompatibility for a number of tissue types. Several aspects have been considered, including wound healing patches [266], bioresorbable sutures [267,268], drug delivery [269], as well as in scaffold development [257] for tissue engineering applications [270,271]. These applications mostly benefit from the elastomeric property of PHAs, especially mcl-PHAs [270,272,273]. Due to its biocompatibility and bioresorbability, PHA is actively involved in multiple research areas.

Shishatskaya et al. [274] utilized poly(3-hydroxybutyrate-*co*-4-hydroxybutyrate) or P(3HB-*co*-4HB) copolymer films, noting the efficacy of the film in terms of reducing inflammation and promoting angiogenesis in the healing process. Meanwhile, the development of PHA-based sutures involving poly(3-hydroxybutyrate-*co*-3-hydroxyvalerate) or P(3HB-*co*-3HV) exhibited similarity in tissue healing response and were comparatively better, as compared to silk-based sutures in intramuscular implantation [268]. A more recent study developed a wound dressing material that incorporated antibiofilm proteins onto P(3HB-*co*-4HB) membranes, hindering bacterial infection on the wound surface [266]. Several modification attempts focusing on wound healing applications using a PHA blend with a synthetic polymer have also been explored [275], involving the enhancement of hydrophilicity, for e.g., a blend of polyvinyl alcohol with P(3HB) was used to produce electrospun fibre mats, which allowed proliferation of human keratinocytes and dermal fibroblasts [276]; introduction of antimicrobial groups; and polyethene glycol methacrylate (PEGMA) in poly-ε-caprolactone was blended with mcl-PHA through enzymatic functionalisation for a topical wound healing patch [277].

The combination of biocompatibility and good mechanical properties is ideal for a material to be considered as a tissue engineering material and PHAs have both. Following tailoring with appropriate processing techniques, PHA has been shown to facilitate cell seeding, adhesion, proliferation, differentiation, and de novo tissue regeneration [278]. In terms of providing physiological support, PHA is known as an excellent tissue scaffold (most notably for bone tissue engineering) [279]. PHBV-hydroxyapatite composites, for instance, have comparable physical and chemical similarities with human bones and serve as an excellent implant candidate for bone scaffolds [280,281]. Not limited to that, composite development of PHAs with bioglass also draws interest in the effort of perfecting the material for bone tissue engineering [282,283], as well as for PHA-bioceramic composite for bone drug delivery [284].

Besides bone tissue, PHAs have also been involved in several other types of tissue engineering scaffold development, especially in soft tissue engineering, including heart valves [285], blood vessel [286], tendon [287], and nerves [288]. Soft tissue engineering for cardiac muscle regeneration, developed using poly(3-hydroxyoctanoate), P(3HO), successfully mimic the mechanical properties of myocardial muscle and claimed to be as good as collagen, which furthers the potential of developing a cardiac patch [289]. In promoting nerve regeneration, PHAs have been used for the production of a bioresorbable conduit. This involved the blending of the crystalline P(3HB) and amorphous P(3HO). The blend percentage is specified—higher P(3HO) content had more correspondence to the peripheral nerves, especially for Young’s modulus and tensile strength [290]. The elastomeric property of PHAs is also useful in the effort of manufacturing matrix material for skin regeneration and wound healing. P(3HO) when combined with bioactive glass nanoparticles exhibited the ability to promote vascularization and exhibited antibacterial properties with enhanced hydrophilicity for skin tissue engineering [273]. Similar composite development strategies have demonstrated similar results but on using P(3HB) instead [291].

Another aspect in utilising PHA as a biomedical material is the development of a PHA-based drug delivery material. PHA was used to encapsulate a drug for controlled drug delivery, with the aim to adjust the material degradation rate over time to control the release kinetics of the compound [267,292,293]. In terms of encapsulation efficiency, the development of nanoparticles to encapsulate the anticancer drug, docetaxel, exploited the hydrophobicity of P(3HB) coupled with poly(lactide-*co*-glycolic) acid, or PLGA. The encapsulation efficiency increased when a higher percentage of PHB was used [294]. Antitumor drug rubomycin successfully promoted tumour inhibition when incorporated into P(3HB) microparticles [295]. Meanwhile, pioneering research involving the P(3HB-*co*-3HV) copolymer used for the encapsulation of ellipticine, an antineoplastic drug, improved drug delivery efficiency two-fold compared to the non-encapsulated drug and exhibited improvement in terms of drug bioavailability at the site [296]. Additional research conjugated poly(2-dimethylaminoethyl methacrylate) and PHA to form thermosensitive and pH-sensitive copolymer constructs for the delivery of doxorubicin, an anticancer drug [297]. Hence, the biocompatibility of PHAs is widely acknowledged and exploited not only as neat polymers but as a part of more complex systems. In the future, it is hoped this family of polymers will have an extensive range of utilisation in tissue engineering and novel future drug development.

#### 2.2.2. Polylactic Acid

**Figure 13:**
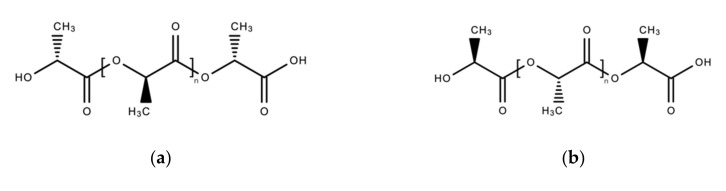

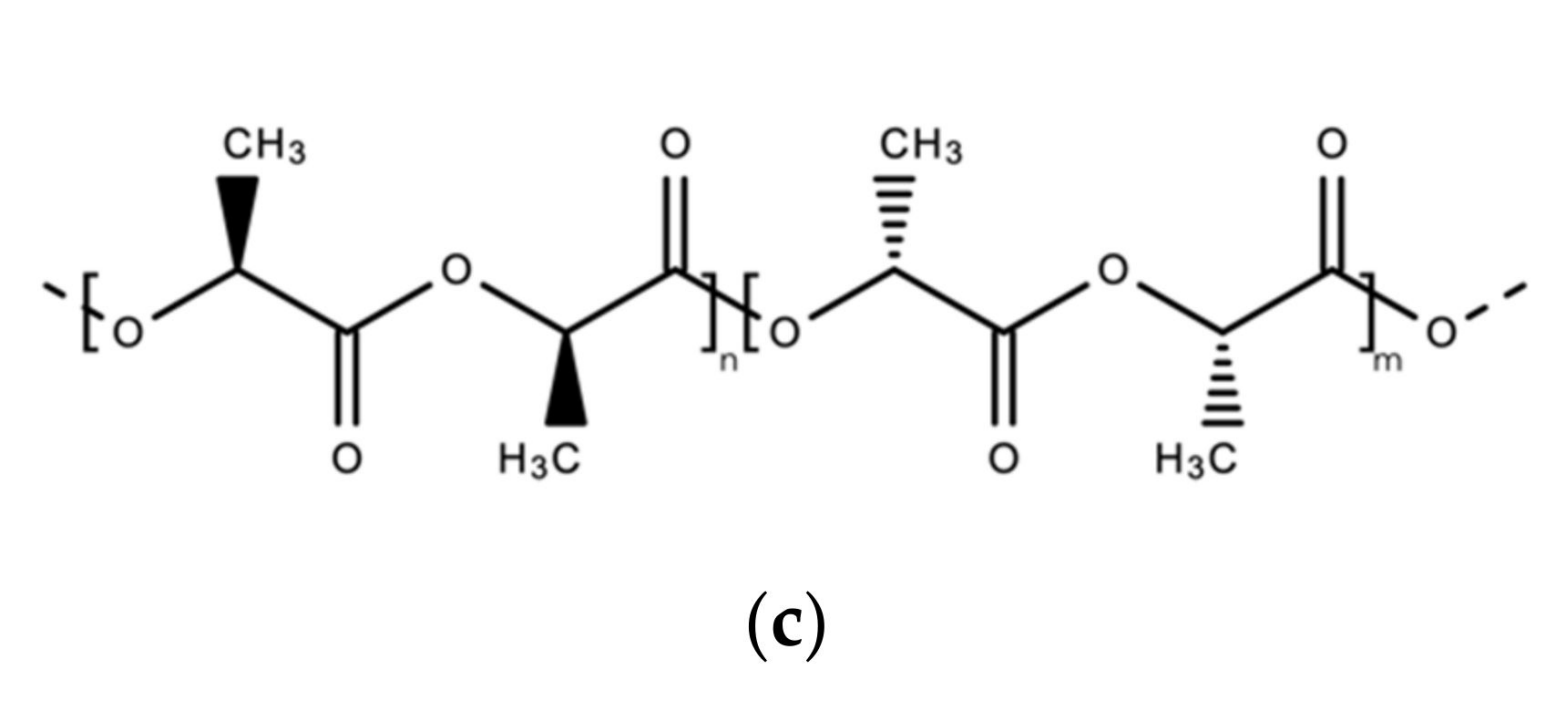
(**a**) PDLA, (**b**) PLLA, and (**c**) PDLLA, where n and m are integers of the repetition units.

Polylactic acid (PLA), or polylactide, is a widely known biopolymer consisting of 2-hydroxypropionic acid or lactic acid repeating units. It is also a polyester consisting of l-lactide and d-lactide, the stereoisomers of PLA. Depending on the type of isomers, three distinct kinds of PLA are known; poly(d-lactic acid) or PDLA, poly(l-lactic acid) or PLLA, and poly(d,l-lactic acid) or PDLLA (Figure 13) [298].

#### Polylactic Variant and Attributes

Commonly, PLA is very brittle and strongly hydrophobic [298]. However, the physical and chemical properties of PLA are also defined by stereoisomeric monomer variation, which was decided based on the isomeric input during synthesis. Optically active d-lactic acid gives a crystallinity characteristic to the polymer matrix; meanwhile, l-lactic acid contributes flexibility [299]. Hence, PDLA is crystalline; PLLA is semicrystalline; and interestingly PDLLA, the polylactic acid polymer with a mixture of both is amorphous in nature. The monomeric composition also defines the thermal properties of PLA, with PDLA and PLLA having a higher decomposition temperature compared to PDLLA. PLA is generally soluble in most organic solvents, but not in aliphatic hydrocarbons and alcohols [298].

#### Production of Polylactic Acid

Whilst PLA itself is not a naturally occurring biopolymer, its monomeric components are found in abundance in nature, mainly produced by lactic acid bacteria, categorised under the Gram-positive bacteria order *Lactobacillales* that use carbohydrate-containing pyranose and furanose sugars as substrates. Hence, PLA production needs chemical synthesis, which involves synthetic pathways. The chemical synthesis of PLA demands high purity of the substrate, whereas lactic acid fermentation may lead to impure products, which then need further downstream processing [300]. Hence, generally, there are three steps involved in the synthesis PLA: (i) lactic acid production by microbes, (ii) lactic acid purification and production of its dimer (lactide), and (iii) polycondensation of lactides through ring-opening polymerisation [301].

**Figure 14:**
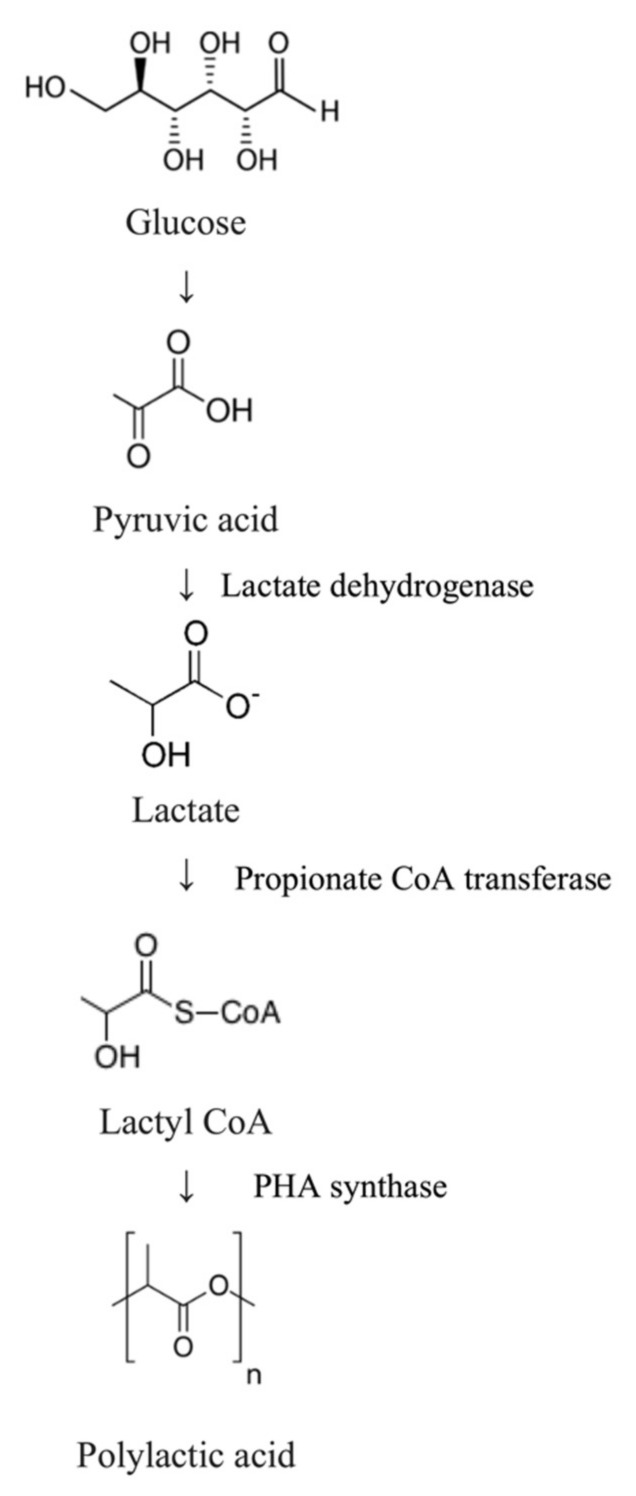
A schematic presentation of the PLA production pathway by recombinant *E. coli*. Adapted with permission from a report by Jung and Lee [304]. Copyright © 2021, Elsevier.

Therefore, scientists have developed an alternative to allow the biosynthesis of PLA by carrying out metabolic engineering. Genetic engineering of *E. coli* by inserting the gene encoding propionate CoA transferase and PHA synthase allowed the recombinant organism to produce PLA from glucose, as conducted by [302]. The glucose molecule is broken down into pyruvic acid, later converted into lactate hydrolysed by lactate dehydrogenase. Then, it is converted to lactyl CoA by propionate CoA transferase and eventually polymerised by the PHA synthase to produce PLA (Figure 14) [303]. The work also interestingly observed the production of PHB-LA copolymer, poly(3-hydroxybutyrate-*co*-lactic acid) by a similar *E. coli* mutant strain, by adding 3-hydroxybutyric acid as a co-feeding material [304].

#### Polylactic Acid in Biomedical Application

PLA is widely known for its potential in biomedical applications. It is an excellent candidate due to its tailorable biodegradability and biocompatibility. Since PLA is a polyester, the ester linkage within the polymer backbone can hydrolyse easily, even without enzymatic action [305]. Due to this degradability, PLA is bioresorbable, allowing the material to naturally disintegrate as the target site is healing [306]. This characteristic is useful and leads to the utilisation of the polymer in a wide range of applications, especially as scaffolds for tissue engineering application and bone fixation purposes. In addition, PLA is a prospective drug delivery material due to its tailorable porosity for controlled adsorption and drug release [307].

In the utilisation of PLA, monomer composition is crucial for the development of the polymer suitable for specific application. PDLLA is a less crystalline polymer and possesses an improved biodegradability for extensive applications in the biomedical area. For example, composite pins made partly of PDLLA were compared with hydroxyapatite pins that were commonly used in bone grafting, and the performance quality observed was similar [308]. On the other hand, PLLA, which has a higher rigidity, is preferable in bone fixture applications in the form of screws or scaffolds [309]. The rate of reabsorption is also relatively longer for more than four years to allow enough time for healing before complete resorption [310]. In another comparative study, the performance of bioresorbable PLLA was compared with a titanium fixture for rabbit mandibular fracture repair and the former showed similar results in terms of mechanical support and healing [311]. PLA can also be modified to adapt to specific applications; for example, PLLA is typically blended with polyglycolic acid for a fixture device [311], and also with hydroxyapatite as a composite bone scaffold material [310].

PLA is also regarded as a potential functional material in drug delivery systems. Preparation of PLA-based drug delivery methods include emulsification, nanoprecipitation, salting-out, spray-drying, and stable dispersion to achieve nano- and microparticles [312]. Incorporation of PLA actually serves as a biodegradable component since it is the most common FDA-approved biopolymer in many drug delivery systems [307]. In order to enable a tailored application, a composite is favoured over a single-material system. For instance, a specialised drug delivery component, D-α-tocopherol polyethene glycol 1000 succinate-polylactide with galactosamine, was developed for targeting liver cancer cells [313]. In another study, PLA-chitin blend microspheres were developed to carry proteins with tailorable degradation rate [314]. Meanwhile, brain targeted nano-carriers integrated with PLA, such as polyethene glycol/PLA with a lactoferrin conjugate, encouraged drug uptake by brain cells [315]. In addition, penetratin-conjugate with similar polyethene glycol/PLA blends enhanced accumulation via endocytosis and direct translocation [316].

One major challenge in the PLA blending is the phase separation between PLA and the component of interest [307]. The immiscibility has disadvantages in collective physical integrity and low mechanical properties in terms of structural strength [317]. However, this situation nevertheless potentially opens up potentialities for exploring the chemistry and molecular interaction of PLA blends for effective composite production for more advanced and extended biomedical applications in the future. For example, an encapsulation strategy in drug delivery material development involving a PLA copolymer, PLGA, was successfully done using titanium dioxide-oleic acid (TiO_2_-OA) by thermally induced phase separation technique or TIPS to become a scaffold with drug release ability [318]. The table summarizing polyester production and biomedical applications can be found in Table 3.

### 2.3. Polyamide

Polyamines are structurally similar to proteins and hence represent the products of the commonest metabolic processes in an organism and can also be commonly derived from bacterial fermentation.

#### 2.3.1. ε-Poly-l-Lysine

**Figure 15:**
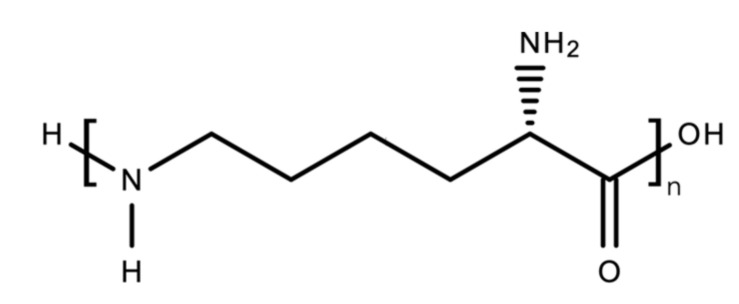
The polymeric structure of ε-poly-l-lysine, where n is integer of the repetition units.

The first polyamine to be discussed here is ε-poly-l-lysine, or ε-PL. Despite lysine’s initial isolation from milk in 1889 [319], lysine in its polymeric form was not discovered until 1977, when Shima and Sakai [320] announced their discovery of what they called the “lysine polymer”, isolated from the culture filtrate of a bacterial strain similar to *Streptomyces albulus* [320,321]. This alkaloid polymer structure is characterised by the l-chirality of the constituent amino acid and by the position of the peptide bond, connecting lysine’s carboxylic acid group, and its ε-amine group (Figure 15) [321,322]. However, despite their successful identification of this novel biopolymer, Shima and Sakai [320] could not determine the physiological function of ε-PL, though contemporary research has now suggested that ε-PL confers antimicrobial and acid-stress protection in ε-PL accumulating genera [323]. Nevertheless, humans have used its chemical, physical, and biological properties for a range of diverse applications in the intervening decades.

#### Current Properties and Subsequent Applications

The physicochemical properties of ε-PL make it well suited to both food industry-related and biomedical applications. Shima and Sakai [320] determined that ε-PL is strongly cationic in solution, owing to the presence of a functional amine group. In fact, [324] proposes ε-PL as a cationic antimicrobial peptide. Cationic materials generate antimicrobial effects due to their interactions with the anionic bacterial membrane, allowing penetration into the bacterial lipid membrane. After a certain threshold concentration is reached, lipid solubilization initiates the break-up of the cell [325]. This property has been confirmed in vitro against *E. coli* and *Listeria innocua*, leading to the inclusion of epsilon poly-l-lysine in a number of studies investigating antimicrobial biomaterials [326]. Xu et al. [327] successfully used ε-PL as an antimicrobial paint against *E. coli* and Methicillin Resistant *Staphylococcus aureus* (MRSA), reducing bacterial load on titanium surfaces implanted into a rodent model by up to Log 3. Moreover, multiple studies have incorporated their ε-PL into hydrogel networks, allowing for flexible, therapeutic factor or cell-loaded antimicrobial materials with self-healing capability [328,329]. Given this unique set of chemical and biological properties, ε-PL has been proposed as a novel antimicrobial wound dressing. Yang et al. [330] combined the antimicrobial properties of silver, chitosan (another cationic polymer), and ε-PL to generate highly biocompatible wound dressings capable of maintaining tissue bed moisture, excellent hygroscopicity, and antimicrobial activity against *E. coli* and *S. aureus*. Although no clinical trials have taken place yet featuring wound dressings impregnated with ε-PL, its antimicrobial properties against both Gram-positive and Gram-negative bacteria, as well as its biocompatibility for wound dressing applications, could lead to novel dressings for the treatment of infected wounds.

**Figure 16:**
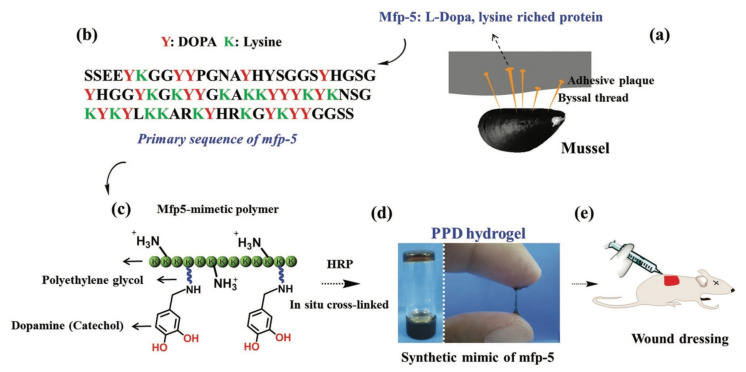
(**a**) Diagram of mussel attachment, polylysine is contained with the plaque; (**b**) the primary amino acid sequence of mfp-5; (**c**) Conjugation of dopamine onto the mfp-5 mimetic polymer; (**d**) Horseradish Peroxidase (HRP) crosslinking of the polymer to form a hydrogel; and (**e**) application of polymer onto a mouse wound model. Used with permission from a report by Wang et al. [333] © 2021 WILEY-VCH Verlag GmbH & Co. KGaA, Weinheim.

Although ε-PL is a polymer, its relatively limited chain length (25–35 amino acid residues) has implications on its physical properties [331]. Despite sparse literature on mechanical properties, the medical applications it is involved in would indicate they are unsuitable for high-strength applications. Indeed, attempts to construct polyglutamic acid (PGA)/ε-PL films for probiotic packaging resulted in decreased construct tensile strength with increasing ε-PL, beyond 2 wt% of ε-PL, despite the elasticity of the developed material greatly increasing with wt% ε-PL [331]. Conversely, research conducted on strongly adhesive mussel foot proteins (Mfps) found that many of the proteins contain (amongst other amino acids) lysine residues. These mfps can adhere to wet, polar surfaces through covalent bond formation, metal chelation, and water displacement, which makes them suitable for applications such as wound adhesives and dressings (Figure 16) [332].

Li et al. [334] constructed Mfp-inspired ε-poly-l-lysine adhesives that were able to resist 100 KPa of shear force between the collagen sheets [334]. Liu et al. [335] demonstrated the biomaterial applications of this adhesive, generating a ε-PL/HA, bioresorbable, antibiotic dressing as an alternative to fibrin glue-requiring dressings. Although hard tissue engineering may be beyond the scope of biomaterials containing ε-poly-l-lysine as the bulk material, research suggests that both soft tissue engineering and mechanically stable materials with polymer coating are niche applications for this polymer. In addition, the antimicrobial and wound-healing biomaterial sectors are highly suitable applications for ε-PL.

Though wound healing is one of the main applications of ε-poly-l-lysine, research has also focused on therapeutic gene and drug delivery. De Smedt et al. [336] discussed the importance of cationic polymers in DNA binding, explaining that cationic molecules encourage polyplex (small DNA strands) condensation, giving researchers a stable gene encapsulation platform. Biodegradable polyethene glycol/polyleucine/ε-PL micelles have been used to successfully transfect 293T kidney fibroblasts with a vector plasmid. Deng et al. [337] noted increased DNA condensation and complex stability enabled by the hydrophobic nature of the chosen polymer. Despite limited clinical trials, stable targeted factor delivery is preferred in cancer treatment, where the need to deliver potentially toxic chemicals, whilst mitigating healthy tissue necrosis, is paramount [338]. Guo et al. [339] developed a targeted system using ε-poly-l-lysine conjugated with a pH sensitive compound that detected the increased acidity of the tumour microenvironment. ε-PL ensures the stability of the system until it reaches its target, allowing maximum drug delivery with limited pathology [339]. The stability of ε-PL allows previously fragile cancer therapeutic structures to survive more rigorous environments. El Assal et al. [340] preserved cancer hunting Natural Killer (NK) cells using ε-PL, where previous cryopreservation attempts using solely contemporary cryoprotectants caused significant cell death. Hence, the bioresorbable property and stabilising qualities of ε-poly-l-lysine have resulted in its application in the fields of cancer and wound healing.

#### Production of the Polymer by Bacteria

Bacterially derived ε-poly-l-lysine has been used as a preservative for more than 15 years, with ε-PL receiving its FDA “GRAS” rating in 2004 [341,342]. The industrial strain of *Streptomyces albulus* soil has advanced from its original soil isolate [320], The isolation of strains producing up to four times the original yield obtained in the 1977 study have been noted in literature [340,343]. Further gains have been made using “genome shuffling”, a technique involving the fusion of bacterial protoplasts generating new genome combinations [344]. This was carried out by Li et al. [345] to almost double the existing yield of *Streptomyces graminearus* cultures. Induced mutation has also been trialled. Optimisations to the growth conditions are also ongoing. They noticed a yield-limiting rise in toxic ROS in the reaction vessel, supplemented their feedstock with astaxanthin, an antioxidant blackcurrant derivative, increasing their yield by 30% [345,346]. A continuous problem with improvements to the *Streptomyces* sp. growth model has been the historical lack of data on the genes expressing ε-PL synthases, a problem which (exacerbated by a lack of ε-PL accumulating organisms) prevented genetic transfer into more industrially suited micro-organisms [321]. However, Yamanaka et al. [347] finally identified the first ε-PL synthase, a non-ribosomal peptide synthase, which was followed up by the first heterologous expression in another Streptomyces species (*Streptomyces lividans*). Moreover, until recently, ε-poly-l-lysine had only been reported in a few strains of *Streptomyces* sp. However, a recent study published in October 2020 by Samadlouie et al. [348] demonstrated for the first time the manufacture of ε-PL in another genus, *Lactobacillus* [321]. Whilst strains of *Lactobacillus delbrueckii* only produced 200 ppm of ε-PL in its growth medium, the future successful translocation of the ε-PL genes into more commonly cultured species may allow manufacture of ε-PL in higher yields.

#### 2.3.2. Poly-γ-Glutamate (γ-PGA)

Similar to ε-poly-l-lysine, poly-γ-glutamate (γ-PGA) is classified as a homo polyamide made up of the repeating subunits of one amino acid residue (glutamate in the case of PGA) [349]. A non-essential amino acid, glutamate can be synthesised de novo in (amongst others) glial cells, indicating its importance in physiological function like neurotransmission [350]. More importantly, glutamate is harmlessly metabolized in the body during the TCA cycle [351]. However, both ε-PL and γ-PGA biopolymers are only bio-resorbed when they contain residues in the L-conformation; the enantiomeric selectivity of their respective enzymes prevents metabolism of the d-conformation of the polymer [352].

#### Properties of γ-PGA

**Figure 17:**
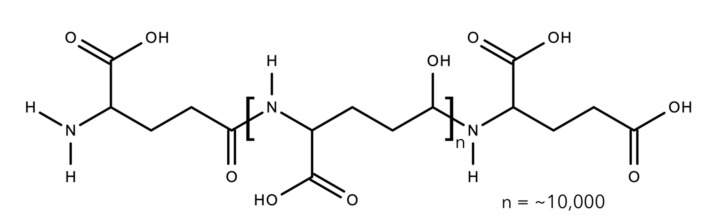
Molecular structure of γ-PGA (note the n number and similarity to nylon).

Chemically γ-PGA and ε-PL have fairly similar structures. Like ε-PL, γ-PGA is an amino acid, with a carboxylic acid, amine, and an *R* group [353]. γ-PGA differs from ε-PL by the presence of another carboxylic acid group (Figure 17), decreasing both the pH of solutions containing γ-PGA in its protonated state and increasing water solubility of the salt (the acid form is insoluble in water due to its propensity to form hydrophobic alpha-helices through intramolecular hydrogen bonding) [354]. This also affects γ-PGA’s charge whilst in solution; making it anionic [355]. γ-PGA’s anionic nature makes it generally non-conducive to lipid membrane solubilisation.

However, its non-degradation under enzymatic attack makes it an attractive candidate as an antimicrobial biomaterial that is resistant to bacterial protease virulence factors. Su et al. [356] observed an increased killing capacity of common mouthwash compounds against *E. coli*, *S. aureus*, and *P. aeruginosa*, by over 30%, with the addition of γ-PGA. This was reconfirmed by the similar antimicrobial capacity of γ-PGA-conjugated contact lens materials by the same group. Research using photosensitisers for use in photodynamic therapy have also used γ-PGA as a stable release platform [356,357]. This approach was demonstrated by Sun et al. [358], who conjugated their electrospun γ-PGA with a 5,10,15,20-tetrakis(1-methylpyridinium-4-yl)porphyrin tetra (p-toluenesulfonate) (TMPyP) photosensitizer, eradicating *S. aureus* from infected mouse model wounds under red light (650 nm) irradiation.

γ-PGA has also been noted for its biomodulator roles in wound healing. Bae et al. [359] proposed that unnecessary damage following wound formation is caused by immune and tissue-specific matrix metalloproteinase secretion. As an inhibitor, the γ-PGA negated these destructive effects with γ-PGA indirectly increasing cell proliferation and matrix formation through inhibition of the hyaluronidase enzyme [359]. This beneficial pro-inflammatory effect was confirmed in skin wounds by Choi et al. [360], who confirmed that daily topical application of γ-PGA to rat wounds increased Transforming Growth Factor (TGF-β1) signalling, angiogenesis, and re-epithelialization, initiating faster healing compared to saline controls. Although inflammation (of which ROS release is a part) is observed in healthy wound healing, ROS associated cytotoxicity have long been identified as markers of chronic wounds [361]. γ-PGA’s ability to be cross-linked (both by radiation and by chemical cross-linking) [362] and water sorption has allowed its use as a stable-release hydrogel platform for ROS inhibiting factors [363,364]. Zhang et al. [364] successfully used chitosan/γ-PGA hydrogel blends loaded with ROS degrading superoxide dismutase enzyme for this purpose, whilst Stevanović et al. [365] conjugated γ-PGA with PLGA to form antioxidant nanoparticles, which increased the ROS scavenging potential by twofold compared to control cells [364,365]. Pisani et al. [366] confirmed this stability, concluding that their chitosan/γ-PGA/adult fibroblast cell hydrogel bioink provided a good compromise between shear resistance (important keeping cells in the matrix) and shear thinning (which promotes printability and cell viability on printing).

Given the drug delivery potential of hydrogels, it is not surprising that (like many of the polymers in this review) γ-PGA has been trialled for targeted cancer drug and gene delivery. Upadhyay et al. [367] used the hyaluronan-CD44 cancer sensing pathway to develop HA/γ-PGA-derivative doxorubicin loaded polymer beads targeted to cancer cells. Despite cationic structures excellent binding properties with respect to nucleic acid-based structures like DNA and RNA, this strong electrostatic attraction prevents dissociation from carrier medium, promoting poor delivery efficiency. Liao et al. [368] were able to partially counteract this using anionic γ-PGA, with the result of increasing the unpacking efficiency of siRNA in the intracellular cytosol. The addition of anionic structures like γ-PGA (with little penalty to complex formation) allows the production of drug delivery devices with improved carrier stability and delivery efficiency [368].

#### Manufacture of Poly-γ-Glutamate

Although γ-PGA is known as an antibacterial biomaterial, its purpose in nature is quite the opposite. The bacterial γ-PGA capsule promotes immunoisolation of the bacteria from the host immune system, whilst increasing the cytotoxicity of lethal toxin, a virulence factor expressed by *Bacillus anthracis*, from which γ-PGA was first isolated [369,370]. Fortunately, a number of alternative prokaryotic and eukaryotic genera, as collated by Candela and Fouet [371], are capable of γ-PGA biosynthesis. Indeed, commercial exploitation of *Bacillus subtilis* began from the manufacture of the vegetable cheese “Natto”, where γ-PGA has been fermented from soybean since at least 1051 AD [372].

Given its use since ancient times, much work has been undertaken to determine the expression of genes, and operation of enzymes pertaining to γ-PGA manufacture. There are however some unknowns. Luo et al. [373] documents the use of the polyglutamate synthase enzyme complex in an ATP-independent reaction, as well as the role of enzymes that convert one enantiomer to another (for example D, L racemization) during the formation of the γ-PGA chain. However, the study also notes that the exact mechanisms and enzymes behind this is currently unknown [373]. What is known however is that fermentation is relatively easy to achieve; [374] reported initial 18-h fermentations at 39 °C for 18 h under high (up to 90%) humidity, followed by a shorter, colder fermentation step during the stationary phase, controlling carbon, nitrogen, oxygen, and H^+^ availability to induce γ-PGA production. Variations in strain fermentation efficacy promote optimisations; industrial *Bacillus amyloliquefaciens* fermentations now produce more than 65 g/kg of culture media γ-PGA following 60 h fermentation time, whilst *Bacillus licheniformis* A14 strain newly isolated from marine sands was able to produce 37 g/kg of culture media after just 24 h of fermentation, an increase in efficiency of 45% of the latter over the former [374,375]. Finally, genetic engineering has been employed to increase yield; Cai et al. [376] used gene deletion vectors to remove by-product biosynthetic enzymes and reduce enzymes controlling γ-PGA degradation in *B. amyloliquefaciens* more than doubling their yield (though their insertion of another set of genes responsible for γ-PGA synthetase expression had the opposite effect) [375], whilst Cai et al. [376] overexpressed genes responsible for NADPH generation in *B. licheniformis* WX-02, finding that increasing the metabolic capacity promoted transcription for two key γ-PGA synthetase genes. Taken together, these significant improvements in yield herald both advancements for the commercial exploitation of γ-PGA in the food industry and the availability of γ-PGA for biomaterials research and implementation. The table summarizing polyamide production and biomedical applications can be found in Table 4.

### 2.4. Polyanhydride

#### 2.4.1. Polyphosphate

**Figure 18:**
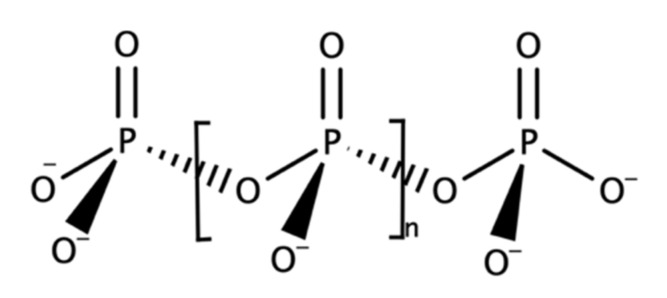
Basic molecular structure of polyphosphate, where n is the number of repeating units.

Polyphosphate is an inorganic polymer, naturally occurring in a wide range of living organisms, including bacteria. It consists of repeated units of phosphate groups (Figure 18). It was isolated in 1890 by Liebermann from Baker’s yeast cells, *Saccharomyces cerevisiae*, originally naming it metaphosphoric acid [377]. Inorganic polyphosphate is typically available in most cell lineages [378], as well as being available in cells as an energy source in the form of ATP. Polyphosphate kinase is the enzyme responsible for the production of inorganic polyphosphate by polymerising terminal phosphate from ATP [379,380].

There are several types of polyphosphates available in nature based on both chain length and molecular pattern. The length of a polyphosphate influences its behaviour in the physiological environment; short chain polyphosphate is acid-soluble, while longer chains are progressively less soluble [378]. This characteristic was discovered primarily through analysis of the plant [381] and yeast [382] derived polymer. Variance in polyphosphate residue length can range from between two to 10 residues for short chain polyphosphate, compared to 500 residues for the long-chain polymer [383]. Conversely, the molecular pattern is usually readily available in a linear form (though tri, tetra, and hexa-cyclic forms have been reported) [384]. Hyperbranched polyphosphate does not naturally occur; this synthetic form of polyphosphate is produced mainly for potential drug delivery vehicle research [385,386,387]. Analytical extraction from a microbial source is typically carried out initially using trichloroacetic acid or perchloric acid as a chaotropic agent for cell disruption, subsequently adding a strong base such as sodium perchlorate or sodium hydroxide to dissolve polyphosphate. Later, dissolved polyphosphate is recovered by alcohol or Ba^2+^ precipitation [382].

#### Microbial Production of Polyphosphate

Generally, the enzyme that is responsible for the synthesis of polyphosphate is polyphosphate kinase [388]. There are two types of polyphosphate kinases: polyphosphate kinase 1 and 2 (PPK1 and PPK2). Prokaryote polyphosphate is typically accumulated intracellularly by PPK1. For instance, PPK1 expression by *E. coli* catalyses the reversible polyphosphate synthesis by extracting terminal phosphate from ATP [389]. PPK2 differs in polyphosphate source, using either guanosine triphosphate (GTP) or ATP for polyphosphate synthesis [390]. Additionally, enzyme stimulation is mediated by polyphosphate, taking the polymer as a donor to commence different enzymatic processes akin to nucleoside diphosphate kinase. The process converts guanosine diphosphate (GDP) into GTP, as ascertained in the metabolic pathways of *P. aeruginosa* [390,391]. In eukaryotic cells such as *S. cerevisiae*, vacuolar transporter chaperone 4 (VT4) availability enables polymerisation of phosphates into polyphosphate and simultaneous transportation into the vacuole, as a reserve for homeostatic balance, including phosphate sequestering, chelating toxic metals, and source of phosphate for DNA replication [392].

Polyphosphate-producing strains such as *Citrobacter freundii* and *Candidatus accumulibacter* have also found roles in the removal of excess polyphosphates in wastewater systems. Genetic modification approaches upon the bacteria enable them to take part in enhanced biological phosphorus removal (EBPR). The modification promotes high efficiency of phosphate uptake and polymerisation in the activated sludge system, more than the amount of phosphate required for the bacterial growth [393,394].

#### Polyphosphate Role in Physiological Processes

The recognition of polyphosphate as one of the critical molecules that affect metabolic pathways in mammalian cells opens up the potential of the development of unique pharmaceutical and therapeutic material, especially related to cellular dysregulation. Currently, it is known as a metabolic fuel in transferring polyphosphate extracellularly to affect intracellular pathways, and also amplifying mitochondrial ATP production [395]. On the other hand, long-chain polyphosphate also promotes antibacterial and antiviral activity by forming ionic bonds with free Mg^2+^ and Ca^2+^, suppressing bacterial and viral viability [396]. The exhaustion of these cations has been shown to retard microbial growth observed in *Bacillus cereus* [397] and *S. aureus* 196E [398]. Besides that, polyphosphate ability to maintain the structural integrity of proteins in their folding mechanism in the presence of stress has been acknowledged [392].

There are a number of physiological pathways that polyphosphate could possibly involve in targeting human cells. In physiological blood clotting mechanisms, long-chained (more than 500 units) polyphosphate with more than 500 phosphate units increases the sensitivity of the contact pathway by increasing the fibrin clot turbidity, whilst the shorter chain (less than 100 units) polymer accelerates the activation of factor V, which inhibits anti-coagulation pathways and promotes clotting [399,400,401]. Moreover, insoluble polyphosphate expressed on platelets has also been linked with the activation of coagulation factor XII and thrombus formation in contact system activation [402]. Polyphosphate also expresses a morphogenic role in bone and cartilage tissue in terms of promoting growth and repair from damages [403]. Apatite or calcium phosphate is a building block of bone tissues in vertebrates, and as such requires a phosphate reservoir for bone maintenance. The suggestion is that polyphosphate has an active part in the calcification process of bone [404], explaining the significant amount of both soluble and insoluble polyphosphates found in osteoblast-like cells [405,406]. Thus, inorganic polyphosphate has many biomedical applications.

## 3. Conclusions

This review has demonstrated the enormous potential of bacteria-derived polymers in biomedical applications. Advances in biochemical engineering methods for optimal bioprocess development, genetic modification methodologies, and artificial selection of microbes are furthering the economic viability of the production of these polymers. Moreover, production of polymers via bacterial fermentation has added advantages, including increased purity, reduced risk of zoonotic infection transmission, the ability to modify feedstock to tune biopolymer properties, the exclusivity of manufacture via only recently determined enzymatic pathways in some microorganisms, and the repeatability of the properties of the manufactured natural polymer. For processes that remain less economically sustainable, the extraordinary variety and promising properties of these biopolymers will certainly encourage in depth research to overcome this hurdle. Hence, bacteria-derived polymers are certainly evolving towards emerging as a family of future sustainable biomedical materials with a huge potential in varied applications, including cancer therapy, wound healing, tissue engineering, medical device development, and drug delivery.

## Figures and Tables

**Table 1:** Summary Table for Bacteria-Derived Polysaccharides.

Polymer	Polymer-Accumulating Bacteria	Biomaterial Properties in Biomedical Application	Ref.
Dextran	*Leuconostoc sp.*, including *L. pseudomesenteroides, L. mesenteroides* and *L. citreum* *Weisella cibaria, Wiesella confusa, Pediococcus pentosaceus*, *Lactobacillus satsumensis*, *and Lactobacillusplantarum*	Incorporation of dextran in drug delivery systems takes advantage of its structural integrity in forming hydrogels Dextran-drug conjugates enhance their analgesic and antipyretic properties whilst reducing their constituent drug’s ulcerogenic effect and also possess anticonvulsant properties Serves as drug carrier material in targeting specific organs	[25,26,27,28,29,30,31,32,33,34,35,36,37,38,39,40,41,42,43,44,45,46,47,48,49,50,51,52,53,54,55,56,57,58,59]
Glycogen	Genera *Streptomyces*, *Rhizobium*, *Methanococcus*, *Streptococcus*, *Enterobacter*, *Escherichia*, *Synechococcus*, *Micropruina* and *Candidatus*	Tissue engineering applications, as a crosslinker for hydrogels, allow for the generation of multifunctional and self-healing biomaterials Shown to increase elongation at break of polymer structures (at the expense of tensile strength) Controlled-release drug delivery has been trialled, especially in anti-cancer therapies	[60,61,62,63,64,65,66,67,68,69,70,71,72,73,74,75,76,77,78,79,80,81,82,83,84,85,86,87,88,89,90,91,92,93,94,95,96,97,98,99,100,101,102,103,104,105,106,107,108,109,110,111,112,113,114,115]
Alginate	Wild-type Alginate Expressors include *Pseudomonas aeruginosa* and *Azotobacter vinelandii* Recombined into *Escherichia coli*	Facilitate appropriate wound moisture retention and wound healing Excellent cell-adhesive and degradation behaviour Successfully used as a minimally invasive delivery system Exceptional sustained release and swelling degree Bind with divalent cations to form crosslinks and susceptible to modifications for tissue engineering applications	[116,117,118,119,120,121,122,123,124,125,126,127,128,129,130,131,132,133,134,135,136,137,138,139,140]
Hyaluronic acid	First commercial production in *Streptococcus zooepidemicus* Genera *Streptococcus* and *Pasteurella*	Swelling ability has found use both in hydrogel tissue engineering research and in contemporary plastic surgery polymer expanding filling materials Synergistic lubricative ability has been trialled for the treatment of joint based pathology such as osteoarthritis HA is effective as an immunoisolation material, with avenues in type 1 diabetes treatment Natural affinity for some cancer surface proteins, such as CD44, promoting a drug delivery role	[141,142,143,144,145,146,147,148,149,150,151,152,153,154,155,156,157,158,159,160,161,162,163,164,165,166,167,168,169,170,171,172,173,174,175,176,177,178,179,180,181,182,183,184,185,186]
Gellan	*Sphingomonas elodea*, *Sphingomonas paucimobilis* and *Pseudomonas elodea*	Forms stable and self-supporting hydrogel and used as a culture media additive Generally used for oral formulations, as gels or coatings of capsules Protect bioactive molecules from the low pH Mostly applied in nasal, ocular, gastric pharmaceutical delivery systems, and as freeze-dried scaffold or sponges in tissue regeneration	[187,188,189,190,191,192,193,194,195,196,197,198,199,200,201,202,203,204,205]
Xanthan	Primarily expressed in *Xanthamonas sp.*, *Xantamonas campestris*	Resist enzymatic digestion in the stomach or small intestine encouraging in colon and stomach delivery systems Improved drug permeation and bioavailability with nasal gels Excellent biomimicking potential Potential biomolecules and therapeutic carriers because of their stability, protection, and controlled release kinetics	[206,207,208,209,210,211,212,213,214,215,216,217,218,219,220,221,222,223,224,225,226,227,228,229,230,231]
Curdlan	First extracted from *Alcaligenes faecalis* var. *myxogenes,* (later reclassified as *Agrobacterium sp.)*	Used as a biological response modifier because of their immunostimulatory properties, anti-infective, and anti-inflammatory Encapsulation vehicle for carrying drugs and other molecules	[232,233,234,235,236,237,238,239,240,241,242,243,244,245,246,247]

**Table 2:** Examples of different aliphatic monomer side chains and the types of PHAs.

*x*	*R*	Polymer Name	Abbreviation	Type
1	methyl	Poly-3-hydroxybutyrate	P(3HB)	scl
	ethyl	Poly-3-hydroxyvalerate	P(3HV)	scl
	propyl	Poly-3-hydroxyhexanoate	P(3HHx)	mcl
	pentyl	Poly-3-hydroxyoctanoate	P(3HO)	mcl
	nonyl	Poly-3-hydroxydodecanoate	P(3HDD)	lcl
2	H	Poly-4-hydroxybutyrate	P(4HB)	scl
	methyl	Poly-3-hydroxyvalerate	P(4HV)	scl
3	H	Poly-5-hydroxyvalerate	P(5HV)	scl
	methyl	Poly-5-hydroxyhexanoate	P(5HHx)	scl
4	hexyl	Poly-6-hydroxydodecanoate	P(6HDD)	mcl

*n* = integer for repeating units.

**Table 3:** Summary Table for Bacteria-Derived Polyesters.

Polymer	Polymer-Accumulating Bacteria	Biomaterial Properties in Biomedical Application	Ref.
Polyhydroxyalkanoates	First isolated from *Bacillus megaterium* Multiple strains of *Bacillus* and *Pseudomonas*, including *P. putida* and *B. aquamaris*	Several aspects have been considered, including wound healing patches by promoting angiogenesis in the healing process, bioresorbable sutures, and in drug delivery with a tailorable material degradation rate Useful in scaffold development for tissue engineering applications, which is biocompatible for a number of tissue types by facilitating cell seeding, adhesion, proliferation, differentiation, and de novo tissue regeneration.	[248,249,250,251,252,253,254,255,256,257,258,259,260,261,262,263,264,265,266,267,268,269,270,271,272,273,274,275,276,277,278,279,280,281,282,283,284,285,286,287,288,289,290,291,292,293,294,295,296,297]
Polylactic acid	PLA monomeric components being synthesized by bacteria of the order *Lactobacillales* Genetically modified *Escherichia coli*	PLA is bioresorbable, allowing the material to naturally disintegrate as the target site is healing Acts as a scaffold for tissue engineering application and bone fixation purposes Prospective drug delivery material due to its tailorable porosity for controlled adsorption and drug release	[298,299,300,301,302,303,304,305,306,307,308,309,310,311,312,313,314,315,316,317,318]

**Table 4:** Summary Table for Bacteria-Derived Polyamides.

Polymer	Polymer-Accumulating Bacteria	Biomaterial Properties in Biomedical Application	Ref.
ε-poly-l-lysine	*Streptococcus albulus* *Streptococcus graminearus* *Lactobacillus delbrueckii*	Cationic properties make ε-poly-l-lysine and excellent antimicrobial biomaterial and DNA binding for future use in gene therapies Has successfully been used as an antibiotic coating on titanium implants Strongly adhesive properties have suggested ε-poly-l-lysine’s role in adhesive wound healing dressings.	[319,320,321,322,323,324,325,326,327,328,329,330,331,332,333,334,335,336,337,338,339,340,341,342,343,344,345,346,347,348]
Poly-γ-glutamate	Multiple strains of genus “*Bacillus”,* including *B. anthracis, B. subtilis, B. licheniformis* and *B. amyloliquefaciens* *B. subtilis* is responsible for most commercial production.	Proposed applications in antimicrobials due to its resistance to protease virulence factors Has improved the bacterial killing capacity of existing and experimental antimicrobials Pro-inflammatory effect may be beneficial in wound healing	[349,350,351,352,353,354,355,356,357,358,359,360,361,362,363,364,365,366,367,368,369,370,371,372,373,374,375,376]

## Data Availability

The data presented in this study are available on request from the corresponding author.

## References

[1] PlasticsEurope (2019). Plastics—The Facts 2019: An Analysis of European Plastics Production, Demand and Waste Data. Belgium. https://www.plasticseurope.org/application/files/9715/7129/9584/FINAL_web_version_Plastics_the_facts2019_14102019.pdf.

[2] Meikle J.L. (1995). American Plastic: A Cultural History.

[3] Potter K.D. (1999). The early history of the resin transfer moulding process for aerospace applications. Compos. Part A Appl. Sci. Manuf..

[4] Hench L.L. (1998). Biomaterials: A forecast for the future. Biomaterials.

[5] Rea S., Bonfield W. (2004). Biocomposites for medical applications. J. Australas. Ceram. Soc..

[6] Kane S.R., Ashby P.D., Pruitt L.A. (2010). Characterization and tribology of PEG-like coatings on UHMWPE for total hip replacements. J. Biomed. Mater. Res. Part A Off. J. Soc. Biomater. Jpn. Soc. Biomater. Aust. Soc. Biomater. Korean Soc. Biomater..

[7] Xing C.-M., Meng F.-N., Quan M., Ding K., Dang Y., Gong Y.-K. (2017). Quantitative fabrication, performance optimization and comparison of PEG and zwitterionic polymer antifouling coatings. Acta Biomater..

[8] Prokop A., Hunkeler D., Powers A., Whitesell R., Wang T. (1998). Water soluble polymers for immunoisolation II: Evaluation of multicomponent microencapsulation systems. Microencapsulation Microgels Iniferters.

[9] Gupta M.K., Walthall J.M., Venkataraman R., Crowder S.W., Jung D.K., Shann S.Y., Feaster T.K., Wang X., Giorgio T.D., Hong C.C. (2011). Combinatorial polymer electrospun matrices promote physiologically-relevant cardiomyogenic stem cell differentiation. PLoS ONE.

[10] Jaidev L., Chatterjee K. (2019). Surface functionalization of 3D printed polymer scaffolds to augment stem cell response. Mater. Des..

[11] Shim W.J., Thomposon R.C. (2015). Microplastics in the ocean. Arch. Environ. Contam. Toxicol..

[12] Astrup T., Fruergaard T., Christensen T.H. (2009). Recycling of plastic: Accounting of greenhouse gases and global warming contributions. Waste Manag. Res..

[13] Pirc U., Vidmar M., Mozer A., Kržan A. (2016). Emissions of microplastic fibers from microfiber fleece during domestic washing. Environ. Sci. Pollut. Res..

[14] Valavanidis A., Iliopoulos N., Gotsis G., Fiotakis K. (2008). Persistent free radicals, heavy metals and PAHs generated in particulate soot emissions and residue ash from controlled combustion of common types of plastic. J. Hazard. Mater..

[15] Rehm B.H. (2010). Bacterial polymers: Biosynthesis, modifications and applications. Nat. Rev. Microbiol..

[16] Inbaraj B.S., Chiu C., Ho G., Yang J., Chen B. (2008). Effects of temperature and pH on adsorption of basic brown 1 by the bacterial biopolymer poly (γ-glutamic acid). Bioresour. Technol..

[17] Lenz R.W., Marchessault R.H. (2005). Bacterial polyesters: Biosynthesis, biodegradable plastics and biotechnology. Biomacromolecules.

[18] Li S.Y., Dong C.L., Wang S.Y., Ye H.M., Chen G.-Q. (2011). Microbial production of polyhydroxyalkanoate block copolymer by recombinant Pseudomonas putida. Appl. Microbiol. Biotech..

[19] Reichmann N.T., Cassona C.P., Gründling A. (2013). Revised mechanism of D-alanine incorporation into cell wall polymers in Gram-positive bacteria. Microbiology.

[20] Chen G.G.-Q. (2009). Plastics from Bacteria: Natural Functions and Applications.

[21] Lopes M.S., Jardini A., Maciel-Filho R. (2012). Poly (lactic acid) production for tissue engineering applications. Proc. Eng..

[22] Tachibana Y., Yamahata M., Kimura S., Kasuya K.-I. (2018). Synthesis, Physical Properties, and Biodegradability of Biobased Poly (butylene succinate-co-butylene oxabicyclate). ACS Sustain. Chem. Eng..

[23] Tarrahi R., Fathi Z., Seydibeyoğlu M.Ö., Doustkhah E., Khataee A. (2020). Polyhydroxyalkanoates (PHA): From production to nanoarchitecture. Int. J. Biol. Macromol..

[24] Morris G., Harding S. (2009). Polysaccharides, microbial. Encyclopedia of Microbiology.

[25] Oliveira J.T., Reis R.L., Reis R.L., Neves N.M., Mano J.F., Gomes M.E., Marques A.P., Azevedo H.S. (2008). 18—Hydrogels from Polysaccharide-Based Materials: Fundamentals and Applications in Regenerative Medicine.

[26] Sajna K.V., Gottumukkala L.D., Sukumaran R.K., Pandey A., Pandey A., Höfer R., Taherzadeh M., Nampoothiri K.M., Larroche C. (2015). Chapter 18—White Biotechnology in Cosmetics. Industrial Biorefineries & White Biotechnology.

[27] Kothari D., Das D., Patel S., Goyal A. (2014). Dextran and food application. Polysacchccharides.

[28] Patel A., Prajapat J. (2013). Food and health applications of exopolysaccharides produced by lactic acid bacteria. Adv. Dairy Res..

[29] Selvi S.S., Eminagic E., Kandur M.Y., Ozcan E., Kasavi C., Oner E.T. (2019). Research and Production of Microbial Polymers for Food Industry. Bioproces. Biomol. Product..

[30] Pasteur L. (1861). On the viscous fermentation and the butyrous fermentation. Bull. Soc. Chim..

[31] Crescenzi V. (1995). Microbial Polysaccharides of Applied Interest—Ongoing Research Activities in Europe. Biotechnol. Progr..

[32] Naessens M., Cerdobbel A., Soetaert W., Vandamme E.J. (2005). *Leuconostoc dextransucrase* and dextran: Production, properties and applications. J. Chem. Technol. Biotech..

[33] Zhou Q., Feng F., Yang Y., Zhao F., Du R., Zhou Z., Han Y. (2018). Characterization of a dextran produced by *Leuconostoc pseudomesenteroides* XG5 from homemade wine. Int. J. Biol. Macromol..

[34] Aman A., Siddiqui N.N., Ul-Qader S.A. (2012). Characterization and potential applications of high molecular weight dextran produced by *Leuconostoc mesenteroides* AA1. Carbohydr. Polym..

[35] Feng F., Zhou Q.Q., Yang Y.F., Zhao F.K., Du R.P., Han Y., Xiao H.Z., Zhou Z.J. (2018). Characterization of highly branched dextran produced by *Leuconostoc citreum* B-2 from pineapple fermented product. Int. J. Biol. Macromol..

[36] Banerjee A., Bandopadhyay R. (2016). Use of dextran nanoparticle: A paradigm shift in bacterial exopolysaccharide based biomedical applications. Int. J. Biol. Macromol..

[37] Maia J., Evangelista M., Gil H., Ferreira L., Gil M.H. (2014). Dextran-based materials for biomedical applications. Carbohydrates Applications in Medicine.

[38] Purama R.K., Arun G. (2005). Dextransucrase production by *Leuconostoc mesenteroides*. Indian J. Microbiol..

[39] Patil S.B., Inamdar S.Z., Reddy K.R., Raghu A.V., Soni S.K., Kulkarni R.V. (2019). Novel biocompatible poly(acrylamide)-grafted-dextran hydrogels: Synthesis, characterization and biomedical applications. J. Microbiol. Meth..

[40] Khalikova E., Susi P., Korpela T. (2005). Microbial dextran-hydrolyzing enzymes: Fundamentals and applications. Microbiol. Mol. Biol. R.

[41] Patel S., Majumder A., Goyal A. (2012). Potentials of Exopolysaccharides from Lactic Acid Bacteria. Indian J. Microbiol..

[42] Baruah R., Maina N.H., Katina K., Juvonen R., Goyal A. (2017). Functional food applications of dextran from *Weissella cibaria* RBA12 from pummelo (*Citrus maxima*). Int. J. Food Microbiol..

[43] Besrour-Aouam N., Fhoula I., Hernández-Alcántara A.M., Mohedano M.L., Najjari A., Prieto A., Ruas-Madiedo P., López P., Ouzari H.-I. (2021). The role of dextran production in the metabolic context of *Leuconostoc* and *Weissella* Tunisian strains. Carbohydr. Polym..

[44] Shukla R., Goyal A. (2013). Novel dextran from *Pediococcus pentosaceus* CRAG3 isolated from fermented cucumber with anti-cancer properties. Int. J. Biol. Macromol..

[45] Wang B., Song Q., Zhao F., Zhang L., Han Y., Zhou Z. (2019). Isolation and characterization of dextran produced by Lactobacillus sakei L3 from Hubei sausage. Carbohydr. Polym..

[46] Freitas F., Torres C.A.V., Reis M.A.M. (2017). Engineering aspects of microbial exopolysaccharide production. Bioresour. Technol..

[47] Gibbons R.J., Banghart S.B. (1967). Synthesis of extracellular dextran by cariogenic bacteria and its presence in human dental plaque. Arch. Oral Biol..

[48] Leathers T.D. (2002). Dextran. Biopolymers.

[49] Rahmat-Zohra R., Waseem S., Aman A., Siddiqui A., Kahkashan-Kazmi S., Rahmat-Zohra R. (2009). Dextran Production by Microbial Biotransformation of Sugarcane Waste. FUUAST J. Biol..

[50] Pescosolido L., Vermonden T., Malda J., Censi R., Dhert W.J.A., Alhaique F., Hennink W.E., Matricardi P. (2011). In situ forming IPN hydrogels of calcium alginate and dextran-HEMA for biomedical applications. Acta Biomater..

[51] Pacelli S., di Muzio L., Paolicelli P., Fortunati V., Petralito S., Trilli J., Casadei M.A. (2020). Dextran-polyethylene glycol cryogels as spongy scaffolds for drug delivery. Int. J. Biol. Macromol..

[52] Redasani V.K., Bari S.B., Redasani V.K., Bari S.B. (2015). Chapter—Approaches for Prodrugs. Prodrug Design.

[53] Praveen B., Shrivastava P., Shrivastava S. (2009). In-Vitro release and pharmacological study of synthesized valproic acid-dextran conjugate. Acta Pharm. Sci..

[54] Cai L.T., Li J.T., Quan S.T., Feng W., Yao J.N., Yang M.L., Li W.Y. (2019). Dextran-based hydrogel with enhanced mechanical performance via covalent and non-covalent cross-linking units carrying adipose-derived stem cells toward vascularized bone tissue engineering. J. Biomed. Mater. Res. Part A.

[55] Jain V., Shukla N., Mahajan S. (2015). Polysaccharides in colon specific drug delivery. J. Transl. Sci..

[56] Hovgaard L., Brondsted H. (1995). Dextran Hydrogels for Colon-Specific Drug-Delivery. J. Control. Release.

[57] Chalasani K.B., Russell-Jones G.J., Jain A.K., Diwan P.V., Jain S.K. (2007). Effective oral delivery of insulin in animal models using vitamin B12-coated dextran nanoparticles. J. Control. Release.

[58] Foerster F., Bamberger D., Schupp J., Weilbacher M., Kaps L., Strobl S., Radi L., Diken M., Strand D., Tuettenberg A. (2016). Dextran-based therapeutic nanoparticles for hepatic drug delivery. Nanomedicine.

[59] Froemel D., Fitzsimons S.J., Frank J., Sauerbier M., Meurer A., Barker J.H. (2013). A Review of Thrombosis and Antithrombotic Therapy in Microvascular Surgery. Eur. Surg. Res..

[60] Manners D.J. (1991). Recent developments in our understanding of glycogen structure. Carbohydr. Polym..

[61] El Khadem H.S., Meyers R.A. (2002). Carbohydrates. Encyclopedia of Physical Science and Technology.

[62] Mischnick P., Momcilovic D., Horton D. (2010). Chemical structure analysis of starch and cellulose derivatives. Advances in Carbohydrate Chemistry and Biochemistry.

[63] Brown A.M., Tekkök S.B., Ransom B.R. (2003). Glycogen regulation and functional role in mouse white matter. J. Physiol..

[64] Hers H.G. (1990). Mechanisms of blood glucose homeostasis. J. Inherit. Metab. Dis..

[65] Fricain J., Granja P., Barbosa M., de Jéso B., Barthe N., Baquey C. (2002). Cellulose phosphates as biomaterials. In vivo biocompatibility studies. Biomaterials.

[66] Klemm D., Schumann D., Udhardt U., Marsch S. (2001). Bacterial synthesized cellulose—Artificial blood vessels for microsurgery. Prog. Polym. Sci..

[67] Torres F.G., Commeaux S., Troncoso O.P. (2012). Biocompatibility of bacterial cellulose based biomaterials. J. Funct. Biomater..

[68] Cifuente J.O., Comino N., D’Angelo C., Marina A., Gil-Carton D., Albesa-Jové D., Guerin M.E. (2020). The allosteric control mechanism of bacterial glycogen biosynthesis disclosed by cryoEM. Curr. Res. Struct. Biol..

[69] Ball S., Colleoni C., Cenci U., Raj J.N., Tirtiaux C. (2011). The evolution of glycogen and starch metabolism in eukaryotes gives molecular clues to understand the establishment of plastid endosymbiosis. J. Exp. Bot..

[70] Adeva-Andany M.M., González-Lucán M., Donapetry-García C., Fernández-Fernández C., Ameneiros-Rodríguez E. (2016). Glycogen metabolism in humans. BBA Clin..

[71] Engelking L.R. (2010). Textbook of Veterinary Physiological Chemistry, Updated 2/e.

[72] Mor I., Cheung E., Vousden K. (2011). Control of glycolysis through regulation of PFK1: Old friends and recent additions. Proc. Cold Spring Harb. Symp. Quant. Biol..

[73] Yamashita T., Ishibashi Y., Nagaoka I., Kasuya K., Masuda K., Warabi H., Shiokawa Y. (1982). Studies on glycogen-induced inflammation of mice. Inflammation.

[74] Zhang J.X., Jones D.V., Clemens M.G. (1994). Effect of activation on neutrophil-induced hepatic microvascular injury in isolated rat liver. Shock.

[75] Ahmed E.M. (2015). Hydrogel: Preparation, characterization, and applications: A review. J. Adv. Res..

[76] Billiet T., Vandenhaute M., Schelfhout J., van Vlierberghe S., Dubruel P. (2012). A review of trends and limitations in hydrogel-rapid prototyping for tissue engineering. Biomaterials.

[77] Zhu J., Marchant R.E. (2011). Design properties of hydrogel tissue-engineering scaffolds. Expert Rev. Med. Devices.

[78] Patra P., Rameshbabu A.P., Das D., Dhara S., Panda A.B., Pal S. (2016). Stimuli-responsive, biocompatible hydrogel derived from glycogen and poly (N-isopropylacrylamide) for colon targeted delivery of ornidazole and 5-amino salicylic acid. Polym. Chem..

[79] Patra P., Patra N., Pal S. (2020). Opposite swelling characteristics through changing the connectivity in a biopolymeric hydrogel based on glycogen and glycine. Polym. Chem..

[80] Evans N.D., Minelli C., Gentleman E., la Pointe V., Patankar S.N., Kallivretaki M., Chen X., Roberts C.J., Stevens M.M. (2009). Substrate stiffness affects early differentiation events in embryonic stem cells. Eur. Cell Mater..

[81] Park J.S., Chu J.S., Tsou A.D., Diop R., Tang Z., Wang A., Li S. (2011). The effect of matrix stiffness on the differentiation of mesenchymal stem cells in response to TGF-β. Biomaterials.

[82] Subramony S.D., Dargis B.R., Castillo M., Azeloglu E.U., Tracey M.S., Su A., Lu H.H. (2013). The guidance of stem cell differentiation by substrate alignment and mechanical stimulation. Biomaterials.

[83] Zhang X., Zhou J., Ying H., Zhou Y., Lai J., Chen J. (2020). Glycogen as a Cross-Linking Agent of Collagen and Nanohydroxyapatite To Form Hydrogels for bMSC Differentiation. ACS Sustain. Chem. Eng..

[84] Schlegel P.N., Group H.S. (2006). Efficacy and safety of histrelin subdermal implant in patients with advanced prostate cancer. J. Urol..

[85] Soon-Shiong P., Heintz R.E., Merideth N., Yao Q.X., Yao Z., Zheng T., Murphy M., Moloney M.K., Schmehl M., Harris M. (1994). Insulin independence in a type 1 diabetic patient after encapsulated islet transplantation. Lancet.

[86] Zhang L., Chen J., Han C. (2009). A multicenter clinical trial of recombinant human GM-CSF hydrogel for the treatment of deep second-degree burns. Wound Repair Regen..

[87] Han Y., Hu B., Wang M., Yang Y., Zhang L., Zhou J., Chen J. (2020). pH-Sensitive tumor-targeted hyperbranched system based on glycogen nanoparticles for liver cancer therapy. Appl. Mater. Today.

[88] Hussain I., Sayed S.M., Liu S., Yao F., Oderinde O., Fu G. (2018). Hydroxyethyl cellulose-based self-healing hydrogels with enhanced mechanical properties via metal-ligand bond interactions. Eur. Polym. J..

[89] Hua J., Ng P.F., Fei B. (2018). High-strength hydrogels: Microstructure design, characterization and applications. J. Polym. Sci. Part B Polym. Phys..

[90] Pourjavadi A., Tavakoli E., Motamedi A., Salimi H. (2018). Facile synthesis of extremely biocompatible double-network hydrogels based on chitosan and poly (vinyl alcohol) with enhanced mechanical properties. J. Appl. Polym. Sci..

[91] Shin H., Olsen B.D., Khademhosseini A. (2012). The mechanical properties and cytotoxicity of cell-laden double-network hydrogels based on photocrosslinkable gelatin and gellan gum biomacromolecules. Biomaterials.

[92] Caballero A., Sulejmani F., Martin C., Pham T., Sun W. (2017). Evaluation of transcatheter heart valve biomaterials: Biomechanical characterization of bovine and porcine pericardium. J. Mech. Behav. Biomed. Mater..

[93] Diba M., Spaans S., Ning K., Ippel B.D., Yang F., Loomans B., Dankers P.Y., Leeuwenburgh S.C. (2018). Self-healing biomaterials: From molecular concepts to clinical applications. Adv. Mater. Interfaces.

[94] Tellado S.F., Balmayor E.R., van Griensven M. (2015). Strategies to engineer tendon/ligament-to-bone interface: Biomaterials, cells and growth factors. Adv. Drug Deliv. Rev..

[95] Antoine A., Tepper B. (1969). Environmental control of glycogen and lipid content of Mycobacterium phlei. Microbiology.

[96] Welles L., Lopez-Vazquez C., Hooijmans C., van Loosdrecht M., Brdjanovic D. (2014). Impact of salinity on the anaerobic metabolism of phosphate-accumulating organisms (PAO) and glycogen-accumulating organisms (GAO). Appl. Microbiol. Biotech..

[97] Zhang C., Chen Y., Liu Y. (2007). The long-term effect of initial pH control on the enrichment culture of phosphorus-and glycogen-accumulating organisms with a mixture of propionic and acetic acids as carbon sources. Chemosphere.

[98] Zhao J., Wang X., Li X., Jia S., Wang Q., Peng Y. (2019). Improvement of partial nitrification endogenous denitrification and phosphorus removal system: Balancing competition between phosphorus and glycogen accumulating organisms to enhance nitrogen removal without initiating phosphorus removal deterioration. Bioresour. Technol..

[99] Preiss J. (1984). Bacterial glycogen synthesis and its regulation. Annu. Rev. Microbiol..

[100] Ali A.A., Shaban K.A., Tantawy E.A. (2014). Effect of poly-β-hydroxybutyrate (PHB) and glycogen producing endophytic bacteria on yield, growth and nutrient. Appl. Sci. Rep..

[101] Birkhed D., Tanzer J. (1979). Glycogen synthesis pathway in Streptococcus mutans strain NCTC 10449S and its glycogen synthesis-defective mutant 805. Arch. Oral Biol..

[102] Braβnta A.F., Eandez C.M., Dáiaaz L.A., Manzanal M.B., Hardisson C. (1986). Glycogen and trehalose accumulation during colony development in Streptomyces antibioticus. Microbiology.

[103] Eydallin G., Montero M., Almagro G., Sesma M.T., Viale A.M., Munoz F.J., Rahimpour M., Baroja-Fernández E., Pozueta-Romero J. (2010). Genome-wide screening of genes whose enhanced expression affects glycogen accumulation in Escherichia coli. DNA Res..

[104] Yu J.-P., Ladapo J., Whitman W.B. (1994). Pathway of glycogen metabolism in Methanococcus maripaludis. J. Bacteriol..

[105] Zevenhuizen L. (1981). Cellular glycogen, β-1, 2-glucan, poly-β-hydroxybutyric acid and extracellular polysaccharides in fast-growing species of Rhizobium. Antonie Van Leeuwenhoek.

[106] He S., McMahon K.D. (2011). Microbiology of ‘*Candidatus* Accumulibacter’in activated sludge. Microb. Biotechnol..

[107] Hickman J.W., Kotovic K.M., Miller C., Warrener P., Kaiser B., Jurista T., Budde M., Cross F., Roberts J.M., Carleton M. (2013). Glycogen synthesis is a required component of the nitrogen stress response in *Synechococcus elongatus* PCC 7942. Algal Res..

[108] Shintani T., Liu W.-T., Hanada S., Kamagata Y., Miyaoka S., Suzuki T., Nakamura K. (2000). *Micropruina glycogenica* gen. nov., sp. nov., a new Gram-positive glycogen-accumulating bacterium isolated from activated sludge. Int. J. Syst. Evol. Microbiol..

[109] Preiss J., Romeo T. (1990). Physiology, biochemistry and genetics of bacterial glycogen synthesis. Advances in Microbial Physiology.

[110] Aikawa S., Nishida A., Ho S.-H., Chang J.-S., Hasunuma T., Kondo A. (2014). Glycogen production for biofuels by the euryhaline cyanobacteria *Synechococcus* sp. strain PCC 7002 from an oceanic environment. Biotechnol. Biofuels.

[111] Brown M.J., Lester J.N. (1980). Comparison of bacterial extracellular polymer extraction methods. Appl. Environ. Microb..

[112] Iglesias A.A., Preiss J. (1992). Bacterial glycogen and plant starch biosynthesis. Biochem. Educ..

[113] Sambou T., Dinadayala P., Stadthagen G., Barilone N., Bordat Y., Constant P., Levillain F., Neyrolles O., Gicquel B., Lemassu A. (2008). Capsular glucan and intracellular glycogen of Mycobacterium tuberculosis: Biosynthesis and impact on the persistence in mice. Mol. Microbiol.

[114] Quilès F., Polyakov P., Humbert F.O., Francius G.G. (2012). Production of extracellular glycogen by Pseudomonas fluorescens: Spectroscopic evidence and conformational analysis by biomolecular recognition. Biomacromolecules.

[115] Celik G.Y., Aslim B., Beyatli Y. (2008). Characterization and production of the exopolysaccharide (EPS) from Pseudomonas aeruginosa G1 and Pseudomonas putida G12 strains. Carbohydr. Polym..

[116] Rehm B.H., Moradali M.F. (2018). Alginates and Their Biomedical Applications.

[117] Bakkevig K., Sletta H., Gimmestad M., Aune R., Ertesvåg H., Degnes K., Christensen B.E., Ellingsen T.E., Valla S. (2005). Role of the Pseudomonas fluorescens alginate lyase (AlgL) in clearing the periplasm of alginates not exported to the extracellular environment. J. Bacteriol..

[118] Hay I.D., Rehman Z.U., Moradali M.F., Wang Y., Rehm B.H. (2013). Microbial alginate production, modification and its applications. Microb. Biotechnol..

[119] Robles-Price A., Wong T.Y., Sletta H., Valla S., Schiller N.L. (2004). AlgX is a periplasmic protein required for alginate biosynthesis in Pseudomonas aeruginosa. J. Bacteriol..

[120] Szekalska M., Puciłowska A., Szymańska E., Ciosek P., Winnicka K. (2016). Alginate: Current use and future perspectives in pharmaceutical and biomedical applications. Int. J. Polym. Sci..

[121] Schmid J., Sieber V., Rehm B. (2015). Bacterial exopolysaccharides: Biosynthesis pathways and engineering strategies. Front. Microbiol..

[122] Nordgård C.T., Nonstad U., Olderøy M.Ø., Espevik T., Draget K.I. (2014). Alterations in mucus barrier function and matrix structure induced by guluronate oligomers. Biomacromolecules.

[123] Powell L.C., Pritchard M.F., Emanuel C., Onsøyen E., Rye P.D., Wright C.J., Hill K.E., Thomas D.W. (2014). A nanoscale characterization of the interaction of a novel alginate oligomer with the cell surface and motility of Pseudomonas aeruginosa. Am. J. Respir. Cell Mol. Biol..

[124] Powell L.C., Sowedan A., Khan S., Wright C.J., Hawkins K., Onsøyen E., Myrvold R., Hill K.E., Thomas D.W. (2013). The effect of alginate oligosaccharides on the mechanical properties of Gram-negative biofilms. Biofouling.

[125] Sun J., Tan H. (2013). Alginate-based biomaterials for regenerative medicine applications. Materials.

[126] Campa C., Holtan S., Nilsen N., Bjerkan T.M., Stokke B.T., SKJåK-BRæK G. (2004). Biochemical analysis of the processive mechanism for epimerization of alginate by mannuronan C-5 epimerase AlgE4. Biochem. J..

[127] Falkeborg M., Cheong L.-Z., Gianfico C., Sztukiel K.M., Kristensen K., Glasius M., Xu X., Guo Z. (2014). Alginate oligosaccharides: Enzymatic preparation and antioxidant property evaluation. Food Chem..

[128] Yang J.-S., Xie Y.-J., He W. (2011). Research progress on chemical modification of alginate: A review. Carbohydr. Polym..

[129] Pawar S.N., Edgar K.J. (2012). Alginate derivatization: A review of chemistry, properties and applications. Biomaterials.

[130] Wong T.W. (2011). Alginate graft copolymers and alginate–co-excipient physical mixture in oral drug delivery. J. Pharm. Pharmacol..

[131] Lee K.Y., Mooney D.J. (2012). Alginate: Properties and biomedical applications. Prog. Polym. Sci..

[132] Bouhadir K.H., Alsberg E., Mooney D.J. (2001). Hydrogels for combination delivery of antineoplastic agents. Biomaterials.

[133] Lucinda-Silva R.M., Salgado H.R.N., Evangelista R.C. (2010). Alginate–chitosan systems: In vitro controlled release of triamcinolone and in vivo gastrointestinal transit. Carbohydr. Polym..

[134] Chang C.-H., Lin Y.-H., Yeh C.-L., Chen Y.-C., Chiou S.-F., Hsu Y.-M., Chen Y.-S., Wang C.-C. (2010). Nanoparticles incorporated in pH-sensitive hydrogels as amoxicillin delivery for eradication of Helicobacter pylori. Biomacromolecules.

[135] Cao L., Mooney D.J. (2007). Spatiotemporal control over growth factor signaling for therapeutic neovascularization. Adv. Drug Deliv. Rev..

[136] Rabbany S.Y., Pastore J., Yamamoto M., Miller T., Rafii S., Aras R., Penn M. (2010). Continuous delivery of stromal cell-derived factor-1 from alginate scaffolds accelerates wound healing. Cell Transplant..

[137] Lópiz-Morales Y., Abarrategi A., Ramos V., Moreno-Vicente C., López-Durán L., López-Lacomba J.L., Marco F. (2010). In vivo comparison of the effects of rhBMP-2 and rhBMP-4 in osteochondral tissue regeneration. Eur. Cell Mater..

[138] Chang S.C., Tobias G., Roy A.K., Vacanti C.A., Bonassar L.J. (2003). Tissue engineering of autologous cartilage for craniofacial reconstruction by injection molding. Plast. Reconstr. Surg..

[139] Zmora S., Glicklis R., Cohen S. (2002). Tailoring the pore architecture in 3-D alginate scaffolds by controlling the freezing regime during fabrication. Biomaterials.

[140] Prang P., Müller R., Eljaouhari A., Heckmann K., Kunz W., Weber T., Faber C., Vroemen M., Bogdahn U., Weidner N. (2006). The promotion of oriented axonal regrowth in the injured spinal cord by alginate-based anisotropic capillary hydrogels. Biomaterials.

[141] Meyer K., Palmer J.W. (1934). The polysaccharide of the vitreous humor. J. Biol. Chem..

[142] Fallacara A., Baldini E., Manfredini S., Vertuani S. (2018). Hyaluronic acid in the third millennium. Polymers.

[143] Atkins E., Sheehan J. (1972). Structure for hyaluronic acid. Nat. New Biol..

[144] Weissmann B., Meyer K. (1952). Structure of hyaluronic acid. The glucuronidic linkage. J. Am. Chem. Soc..

[145] Ward P.D., Thibeault S.L., Gray S.D. (2002). Hyaluronic acid: Its role in voice. J. Voice.

[146] Anilkumar T., Muhamed J., Jose A., Jyothi A., Mohanan P., Krishnan L.K. (2011). Advantages of hyaluronic acid as a component of fibrin sheet for care of acute wound. Biologicals.

[147] Frasca P., Harper R., Katz J. (1981). Scanning electron microscopy studies of collagen, mineral and ground substance in human cortical bone. Scan. Electron. Microsc..

[148] Mathews M.B., Decker L. (1977). Comparative studies of water sorption of hyaline cartilage. Biochim. Biophys. Acta BBA Gen. Subj..

[149] Reddi A., Piez K.A. (1984). Extracellular Matrix Biochemistry.

[150] Hardingham T. (1998). Chondroitin sulfate and joint disease. Osteoarthr. Cartil..

[151] Rosines E., Schmidt H.J., Nigam S.K. (2007). The effect of hyaluronic acid size and concentration on branching morphogenesis and tubule differentiation in developing kidney culture systems: Potential applications to engineering of renal tissues. Biomaterials.

[152] Shi X., Zaia J. (2009). Organ-specific heparan sulfate structural phenotypes. J. Biol. Chem..

[153] Jahn M., Baynes J.W., Spiteller G. (1999). The reaction of hyaluronic acid and its monomers, glucuronic acid and N-acetylglucosamine, with reactive oxygen species. Carbohydr. Res..

[154] Kogan G., Šoltés L., Stern R., Gemeiner P. (2007). Hyaluronic acid: A natural biopolymer with a broad range of biomedical and industrial applications. Biotechnol. Lett..

[155] Greene G.W., Zappone B., Banquy X., Lee D.W., Söderman O., Topgaard D., Israelachvili J.N. (2012). Hyaluronic acid–collagen network interactions during the dynamic compression and recovery of cartilage. Soft Matter.

[156] Lai V.K., Nedrelow D.S., Lake S.P., Kim B., Weiss E.M., Tranquillo R.T., Barocas V.H. (2016). Swelling of collagen-hyaluronic acid co-gels: An in vitro residual stress model. Ann. Biomed. Eng..

[157] Miranda D.G., Malmonge S.M., Campos D.M., Attik N.G., Grosgogeat B., Gritsch K. (2016). A chitosan-hyaluronic acid hydrogel scaffold for periodontal tissue engineering. J. Biomed. Mater. Res. Part B Appl. Biomater..

[158] Sionkowska A., Kaczmarek B., Lewandowska K., Grabska S., Pokrywczyńska M., Kloskowski T., Drewa T. (2016). 3D composites based on the blends of chitosan and collagen with the addition of hyaluronic acid. Int. J. Biol. Macromol..

[159] Beasley K.L., Weiss M.A., Weiss R.A. (2009). Hyaluronic acid fillers: A comprehensive review. Facial Plast. Surg..

[160] Clark C.P. (2007). Animal-based hyaluronic acid fillers: Scientific and technical considerations. Plast. Reconstr. Surg..

[161] Edwards P.C., Fantasia J.E. (2007). Review of long-term adverse effects associated with the use of chemically-modified animal and nonanimal source hyaluronic acid dermal fillers. Clin. Interv. Aging.

[162] Romagnoli M., Belmontesi M. (2008). Hyaluronic acid–based fillers: Theory and practice. Clin. Dermatol..

[163] Raeissadat S.A., Rayegani S.M., Forogh B., Abadi P.H., Moridnia M., Dehgolan S.R. (2018). Intra-articular ozone or hyaluronic acid injection: Which one is superior in patients with knee osteoarthritis? A 6-month randomized clinical trial. J. Pain Res..

[164] Lin W., Liu Z., Kampf N., Klein J. (2020). The Role of Hyaluronic Acid in Cartilage Boundary Lubrication. Cells.

[165] Das S., Banquy X., Zappone B., Greene G.W., Jay G.D., Israelachvili J.N. (2013). Synergistic interactions between grafted hyaluronic acid and lubricin provide enhanced wear protection and lubrication. Biomacromolecules.

[166] Harrington S., Ott L., Karanu F., Ramachandran K., Stehno-Bittel L. (2021). A Versatile Microencapsulation Platform for Hyaluronic Acid and Polyethylene Glycol. Tissue Eng. Part A.

[167] Harrington S., Williams J., Rawal S., Ramachandran K., Stehno-Bittel L. (2017). Hyaluronic acid/collagen hydrogel as an alternative to alginate for long-term immunoprotected islet transplantation. Tissue Eng. Part A.

[168] Resnick N.M., Clarke M.R., Siegfried J.M., Landreneau R., Asman D.C., Ge L., Kierstead L.S., Dougherty G.D., Cooper D.L. (1998). Expression of the cell adhesion molecule CD44 in human lung tumors and cell lines. Mol. Diagn..

[169] Yang B., Yang B.L., Savani R.C., Turley E.A. (1994). Identification of a common hyaluronan binding motif in the hyaluronan binding proteins RHAMM, CD44 and link protein. EMBO J..

[170] Penno M.B., August J.T., Baylin S.B., Mabry M., Linnoila R.I., Lee V.S., Croteau D., Yang X.L., Rosada C. (1994). Expression of CD44 in human lung tumors. Cancer Res..

[171] Lee S.Y., Kang M.S., Jeong W.Y., Han D.-W., Kim K.S. (2020). Hyaluronic Acid-Based Theranostic Nanomedicines for Targeted Cancer Therapy. Cancers.

[172] Li Y., Le T.M.D., Bui Q.N., Yang H.Y., Lee D.S. (2019). Tumor acidity and CD44 dual targeting hyaluronic acid-coated gold nanorods for combined chemo-and photothermal cancer therapy. Carbohydr. Polym..

[173] Wickens J.M., Alsaab H.O., Kesharwani P., Bhise K., Amin M.C.I.M., Tekade R.K., Gupta U., Iyer A.K. (2017). Recent advances in hyaluronic acid-decorated nanocarriers for targeted cancer therapy. Drug Discov. Today.

[174] Yu M., Jambhrunkar S., Thorn P., Chen J., Gu W., Yu C. (2013). Hyaluronic acid modified mesoporous silica nanoparticles for targeted drug delivery to CD44-overexpressing cancer cells. Nanoscale.

[175] Silvipriya K., Kumar K.K., Bhat A., Kumar B.D., John A., Lakshmanan P. (2015). Collagen: Animal sources and biomedical application. J. Appl. Pharm. Sci..

[176] Van Der Laan L.J., Lockey C., Griffeth B.C., Frasier F.S., Wilson C.A., Onions D.E., Hering B.J., Long Z., Otto E., Torbett B.E. (2000). Infection by porcine endogenous retrovirus after islet xenotransplantation in SCID mice. Nature.

[177] Moses A.E., Wessels M.R., Zalcman K., Albertí S., Natanson-Yaron S., Menes T., Hanski E. (1997). Relative contributions of hyaluronic acid capsule and M protein to virulence in a mucoid strain of the group A Streptococcus. Infect. Immun..

[178] Wessels M.R., Moses A.E., Goldberg J.B., DiCesare T.J. (1991). Hyaluronic acid capsule is a virulence factor for mucoid group A streptococci. Proc. Natl. Acad. Sci. USA.

[179] Zeng Y., Zeng W., Zhou Q., Jia X., Li J., Yang Z., Hao Y., Liu J. (2019). Hyaluronic acid mediated biomineralization of multifunctional ceria nanocomposites as ROS scavengers and tumor photodynamic therapy agents. J. Mat. Chem. B.

[180] Gunasekaran V., Gowdhaman D., Ponnusami V. (2020). Role of membrane proteins in bacterial synthesis of hyaluronic acid and their potential in industrial production. Int. J. Biol. Macromol..

[181] Chong B.F., Nielsen L.K. (2003). Aerobic cultivation of Streptococcus zooepidemicus and the role of NADH oxidase. Biochem. Eng. J..

[182] Mohan N., Tadi S.R.R., Pavan S.S., Sivaprakasam S. (2020). Deciphering the role of dissolved oxygen and N-acetyl glucosamine in governing higher molecular weight hyaluronic acid synthesis in Streptococcus zooepidemicus cell factory. Appl. Microbiol. Biotech..

[183] Arslan N.P., Aydogan M.N. (2020). Evaluation of Sheep Wool Protein Hydrolysate and Molasses as Low-Cost Fermentation Substrates for Hyaluronic Acid Production by Streptococcus zooepidemicus ATCC 35246. Waste Biomass Valor..

[184] Chien L.-J., Lee C.-K. (2007). Hyaluronic acid production by recombinant Lactococcus lactis. Appl. Microbiol. Biotech..

[185] Yu H., Stephanopoulos G. (2008). Metabolic engineering of Escherichia coli for biosynthesis of hyaluronic acid. Metab. Eng..

[186] Li Y., Li G., Zhao X., Shao Y., Wu M., Ma T. (2019). Regulation of hyaluronic acid molecular weight and titer by temperature in engineered Bacillus subtilis. 3 Biotech..

[187] Prajapati V.D., Jani G.K., Zala B.S., Khutliwala T.A. (2013). An insight into the emerging exopolysaccharide gellan gum as a novel polymer. Carbohydr. Polym..

[188] Manjanna K. (2010). Natural polysaccharide hydrogels as novel excipients for modified drug delivery systems: A review. Int. J. Chemtech. Res..

[189] Pszczola D.E. (1993). Gellan gum wins IFT’s food technology industrial achievement award. Food Technol..

[190] Zhang H., Zhang F., Yuan R. (2020). Applications of natural polymer-based hydrogels in the food industry. Hydrogels Based on Natural Polymers.

[191] Kang K.S., Veeder G.T., Mirrasoul P.J., Kaneko T., Cottrell I.W. (1982). Agar-like polysaccharide produced by a Pseudomonas species: Production and basic properties. Appl. Environ. Microb..

[192] Morris E.R., Nishinari K., Rinaudo M. (2012). Gelation of gellan—A review. Food Hydrocolloid.

[193] Osmałek T., Froelich A., Tasarek S. (2014). Application of gellan gum in pharmacy and medicine. Int. J. Pharm..

[194] Mahdi M.H., Conway B.R., Smith A.M. (2015). Development of mucoadhesive sprayable gellan gum fluid gels. Int. J. Pharm..

[195] Zia K.M., Tabasum S., Khan M.F., Akram N., Akhter N., Noreen A., Zuber M. (2018). Recent trends on gellan gum blends with natural and synthetic polymers: A review. Int. J. Biol. Macromol..

[196] Bajaj I.B., Survase S.A., Saudagar P.S., Singhal R.S. (2007). Gellan gum: Fermentative production, downstream processing and applications. Food Technol. Biotech..

[197] Bacelar A.H., Silva-Correia J., Oliveira J.M., Reis R.L. (2016). Recent progress in gellan gum hydrogels provided by functionalization strategies. J. Mat. Chem. B.

[198] Novac O., Lisa G., Profire L., Tuchilus C., Popa M. (2014). Antibacterial quaternized gellan gum based particles for controlled release of ciprofloxacin with potential dermal applications. Mater. Sci. Eng. C.

[199] Kumar S., Kaur P., Bernela M., Rani R., Thakur R. (2016). Ketoconazole encapsulated in chitosan-gellan gum nanocomplexes exhibits prolonged antifungal activity. Int. J. Biol. Macromol..

[200] Liu L., Wang B., Gao Y., Bai T.-C. (2013). Chitosan fibers enhanced gellan gum hydrogels with superior mechanical properties and water-holding capacity. Carbohydr. Polym..

[201] Stevens L., Gilmore K.J., Wallace G.G. (2016). Tissue engineering with gellan gum. Biomater. Sci..

[202] Lozano R., Stevens L., Thompson B.C., Gilmore K.J., Gorkin R., Stewart E.M., Panhuis M., Romero-Ortega M., Wallace G.G. (2015). 3D printing of layered brain-like structures using peptide modified gellan gum substrates. Biomaterials.

[203] Meier C., Welland M.E. (2011). Wet-spinning of amyloid protein nanofibers into multifunctional high-performance biofibers. Biomacromolecules.

[204] Da Silva L.P., Cerqueira M.T., Sousa R.A., Reis R.L., Correlo V.M., Marques A.P. (2014). Engineering cell-adhesive gellan gum spongy-like hydrogels for regenerative medicine purposes. Acta Biomater..

[205] Oliveira J.T., Gardel L.S., Rada T., Martins L., Gomes M.E., Reis R.L. (2010). Injectable gellan gum hydrogels with autologous cells for the treatment of rabbit articular cartilage defects. J. Orthop. Res..

[206] Petri D.F. (2015). Xanthan gum: A versatile biopolymer for biomedical and technological applications. J. Appl. Polym. Sci..

[207] Tao F., Wang X., Ma C., Yang C., Tang H., Gai Z., Xu P. (2012). Genome sequence of Xanthomonas campestris JX, an industrially productive strain for Xanthan gum. Am. Soc. Microbiol..

[208] Janse J.D. (2005). Phytobacteriology: Principles and Practice.

[209] Patel J., Maji B., Moorthy N.H.N., Maiti S. (2020). Xanthan gum derivatives: Review of synthesis, properties and diverse applications. RSC Adv..

[210] Garcıa-Ochoa F., Santos V., Casas J., Gómez E. (2000). Xanthan gum: Production, recovery, and properties. Biotechnol. Adv..

[211] Ielpi L., Couso R., Dankert M. (1993). Sequential assembly and polymerization of the polyprenol-linked pentasaccharide repeating unit of the xanthan polysaccharide in Xanthomonas campestris. J. Bacteriol..

[212] Han G., Wang G., Zhu X., Shao H., Liu F., Yang P., Ying Y., Wang F., Ling P. (2012). Preparation of xanthan gum injection and its protective effect on articular cartilage in the development of osteoarthritis. Carbohydr. Polym..

[213] Palaniraj A., Jayaraman V. (2011). Production, recovery and applications of xanthan gum by Xanthomonas campestris. J. Food Eng..

[214] Camesano T.A., Wilkinson K.J. (2001). Single molecule study of xanthan conformation using atomic force microscopy. Biomacromolecules.

[215] Carmona J.A., Lucas A., Ramírez P., Calero N., Muñoz J. (2015). Nonlinear and linear viscoelastic properties of a novel type of xanthan gum with industrial applications. Rheol. Acta.

[216] Junyaprasert V.B., Manwiwattanakul G. (2008). Release profile comparison and stability of diltiazem–resin microcapsules in sustained release suspensions. Int. J. Pharm..

[217] Psomas S., Liakopoulou-Kyriakides M., Kyriakidis D. (2007). Optimization study of xanthan gum production using response surface methodology. Biochem. Eng. J..

[218] Liu Z., Yao P. (2015). Injectable shear-thinning xanthan gum hydrogel reinforced by mussel-inspired secondary crosslinking. RSC Adv..

[219] Bueno V.B., Bentini R., Catalani L.H., Petri D.F.S. (2013). Synthesis and swelling behavior of xanthan-based hydrogels. Carbohydr. Polym..

[220] Hoffman A.S. (2012). Hydrogels for biomedical applications. Adv. Drug Deliv. Rev..

[221] Alvarez-Mancenido F., Landin M., Martinez-Pacheco R. (2008). Konjac glucomannan/xanthan gum enzyme sensitive binary mixtures for colonic drug delivery. Eur. J. Pharm. Biopharm..

[222] Sinha V., Kumria R. (2002). Binders for colon specific drug delivery: An in vitro evaluation. Int. J. Pharm..

[223] Sethi S., Kaith B.S., Kaur M., Sharma N., Kumar V. (2020). Cross-linked xanthan gum–starch hydrogels as promising materials for controlled drug delivery. Cellulose.

[224] Hu X., Wang K., Yu M., He P., Qiao H., Zhang H., Wang Z. (2019). Characterization and Antioxidant Activity of a Low-Molecular-Weight Xanthan Gum. Biomolecules.

[225] Abu-Huwaij R., Obaidat R.M., Sweidan K., Al-Hiari Y. (2011). Formulation and in vitro evaluation of xanthan gum or carbopol 934-based mucoadhesive patches, loaded with nicotine. Aaps Pharmscitech..

[226] Manconi M., Mura S., Manca M.L., Fadda A.M., Dolz M., Hernandez M., Casanovas A., Díez-Sales O. (2010). Chitosomes as drug delivery systems for C-phycocyanin: Preparation and characterization. Int. J. Pharm..

[227] Shiledar R.R., Tagalpallewar A.A., Kokare C.R. (2014). Formulation and in vitro evaluation of xanthan gum-based bilayered mucoadhesive buccal patches of zolmitriptan. Carbohydr. Polym..

[228] Bueno V.B., Bentini R., Catalani L.H., Barbosa L.R., Petri D.F.S. (2014). Synthesis and characterization of xanthan–hydroxyapatite nanocomposites for cellular uptake. Mater. Sci. Eng. C.

[229] Bueno V.B., Takahashi S.H., Catalani L.H., de Torresi S.I.C., Petri D.F.S. (2015). Biocompatible xanthan/polypyrrole scaffolds for tissue engineering. Mater. Sci. Eng. C.

[230] Darzi H.H., Larimi S.G., Darzi G.N. (2012). Synthesis, characterization and physical properties of a novel xanthan gum/polypyrrole nanocomposite. Synth. Met..

[231] Glaser T., Bueno V.B., Cornejo D.R., Petri D.F., Ulrich H. (2015). Neuronal adhesion, proliferation and differentiation of embryonic stem cells on hybrid scaffolds made of xanthan and magnetite nanoparticles. Biomed. Mater..

[232] McIntosh M., Stone B.A., Stanisich V.A. (2005). Curdlan and other bacterial (1→3)-β-d-glucans. Appl. Microbiol. Biotech..

[233] Harada T., Masada M., Fujimori K., Maeda I. (1966). Production of a firm, resilient gel-forming polysaccharide by a mutant of Alcaligenes faecalis var. myxogenes 10 C3. Agric. Biol. Chem..

[234] Zhang R., Edgar K.J. (2014). Properties, chemistry, and applications of the bioactive polysaccharide curdlan. Biomacromolecules.

[235] US Food and Drug Administration (2020). CFR 172-Food Additives Permitted for Direct Addition to Food for Human Consumption: Curdlan.

[236] Steinbüchel A., Hofrichter M., Koyama T., Vandamme E.J., de Baets S. (2002). Biopolymers Online: Biology, Chemistry, Biotechnology, Applications: Polysaccharides 1: Polysaccharides from Prokaryotes.

[237] Funami T., Funami M., Tawada T., Nakao Y. (1999). Decreasing oil uptake of doughnuts during deep-fat frying using curdlan. J. Food Sci..

[238] Yotsuzuka F. (2001). Curdlan. Handbook of Dietary Fiber.

[239] Kasai N., Harada T. (1980). Ultrastructure of Curdlan.

[240] Bohn J.A., BeMiller J.N. (1995). (1→3)-β-d-Glucans as biological response modifiers: A review of structure-functional activity relationships. Carbohydr. Polym..

[241] Vannucci L., Krizan J., Sima P., Stakheev D., Caja F., Rajsiglova L., Horak V., Saieh M. (2013). Immunostimulatory properties and antitumor activities of glucans. Int. J. Oncol..

[242] Stone B., Clarke A. (1992). Chemistry and Biology of (1-3)-β-Glucans.

[243] Kanke M., Tanabe E., Katayama H., Koda Y., Yoshitomi H. (1995). Application of curdlan to controlled drug delivery. III. Drug release from sustained release suppositories in vitro. Biol. Pharm. Bull..

[244] Na K., Park K.-H., Kim S.W., Bae Y.H. (2000). Self-assembled hydrogel nanoparticles from curdlan derivatives: Characterization, anti-cancer drug release and interaction with a hepatoma cell line (HepG2). J. Control. Release.

[245] Delatte S.J., Evans J., Hebra A., Adamson W., Othersen H.B., Tagge E.P. (2001). Effectiveness of beta-glucan collagen for treatment of partial-thickness burns in children. J. Pediatric Surg..

[246] Basha R.Y., Sampath-Kumar T., Doble M. (2017). Electrospun nanofibers of curdlan (β-1, 3 glucan) blend as a potential skin scaffold material. Macromol. Mater. Eng..

[247] Hsieh W.-C., Hsu C.-C., Shiu L.-Y., Zeng Y.-J. (2017). Biocompatible testing and physical properties of curdlan-grafted poly (vinyl alcohol) scaffold for bone tissue engineering. Carbohydr. Polym..

[248] Keshavarz T., Roy I. (2010). Polyhydroxyalkanoates: Bioplastics with a green agenda. Curr. Opin. Microbiol..

[249] Berezina N., Martelli S.M. (2014). Polyhydroxyalkanoates: Structure, properties and sources. RSC Green Chem. Ser..

[250] Martínez-Abad A., Cabedo L., Oliveira C.S., Hilliou L., Reis M., Lagarón J.M. (2016). Characterization of polyhydroxyalkanoate blends incorporating unpurified biosustainably produced poly (3-hydroxybutyrate-co-3-hydroxyvalerate). J. Appl. Polym. Sci..

[251] Tan G.-Y.A., Chen C.-L., Li L., Ge L., Wang L., Razaad I.M.N., Li Y., Zhao L., Mo Y., Wang J.-Y. (2014). Start a Research on Biopolymer Polyhydroxyalkanoate (PHA): A Review. Polymers.

[252] Li L.Z., Huang W., Wang B.J., Wei W.F., Gu Q., Chen P. (2015). Properties and structure of polylactide/poly (3-hydroxybutyrate-co-3-hydroxyvalerate) (PLA/PHBV) blend fibers. Polymer.

[253] Liu Q.S., Zhang H.X., Deng B.Y., Zhao X.Y. (2014). Poly(3-hydroxybutyrate) and Poly(3-hydroxybutyrate-co-3-hydroxyvalerate): Structure, Property, and Fiber. Int. J. Polym. Sci..

[254] Ouyang S.P., Luo R.C., Chen S.S., Liu Q., Chung A., Wu Q., Chen G.Q. (2007). Production of polyhydroxyalkanoates with high 3-hydroxydodecanoate monomer content by fadB and fadA knockout mutant of Pseudomonas putida KT2442. Biomacromolecules.

[255] Bhatia S.K., Gurav R., Choi T.R., Jung H.R., Yang S.Y., Song H.S., Jeon J.M., Kim J.S., Lee Y.K., Yang Y.H. (2019). Poly(3-hydroxybutyrate-co-3-hydroxyhexanoate) production from engineered Ralstonia eutropha using synthetic and anaerobically digested food waste derived volatile fatty acids. Int. J. Biol. Macromol..

[256] Budde C.F., Riedel S.L., Willis L.B., Rha C., Sinskey A.J. (2011). Production of Poly(3-Hydroxybutyrate-co-3-Hydroxyhexanoate) from Plant Oil by Engineered Ralstonia eutropha Strains. Appl. Environ. Microb..

[257] Grande D., Ramier J., Versace D.L., Renard E., Langlois V. (2017). Design of functionalized biodegradable PHA-based electrospun scaffolds meant for tissue engineering applications. New Biotechnol..

[258] Mozejko-Ciesielska J., Szacherska K., Marciniak P. (2019). Pseudomonas Species as Producers of Eco-friendly Polyhydroxyalkanoates. J. Polym. Environ..

[259] Mohapatra S., Maity S., Dash H.R., Das S., Pattnaik S., Rath C.C., Samantaray D. (2017). Bacillus and biopolymer: Prospects and challenges. Biochem. Biophys. Rep..

[260] Basnett P., Marcello E., Lukasiewicz B., Panchal B., Nigmatullin R., Knowles J.C., Roy I. (2018). Biosynthesis and characterization of a novel, biocompatible medium chain length polyhydroxyalkanoate by Pseudomonas mendocina CH50 using coconut oil as the carbon source. J. Mater. Sci. Mater. M.

[261] Basnett P., Lukasiewicz B., Marcello E., Gura H.K., Knowles J.C., Roy I. (2017). Production of a novel medium chain length poly(3-hydroxyalkanoate) using unprocessed biodiesel waste and its evaluation as a tissue engineering scaffold. Microb. Biotechnol..

[262] Lukasiewicz B., Basnett P., Nigmatullin R., Matharu R., Knowles J.C., Roy I. (2018). Binary polyhydroxyalkanoate systems for soft tissue engineering. Acta Biomater..

[263] Le Meur S., Zinn M., Egli T., Thony-Meyer L., Ren Q. (2012). Production of medium-chain-length polyhydroxyalkanoates by sequential feeding of xylose and octanoic acid in engineered Pseudomonas putida KT2440. BMC Biotechnol..

[264] Wang Y., Horlamus F., Henkel M., Kovacic F., Schlafle S., Hausmann R., Wittgens A., Rosenau F. (2019). Growth of engineered Pseudomonas putida KT2440 on glucose, xylose, and arabinose: Hemicellulose hydrolysates and their major sugars as sustainable carbon sources. GCB Bioenergy.

[265] Salvachua D., Rydzak T., Auwae R., de Capite A., Black B.A., Bouvier J.T., Cleveland N.S., Elmore J.R., Huenemann J.D., Katahira R. (2019). Metabolic engineering of Pseudomonas putida for increased polyhydroxyalkanoate production from lignin. Microb. Biotechnol..

[266] Marcano A., Haidar N.B., Marais S., Valleton J.M., Duncan A.C. (2017). Designing Biodegradable PHA-Based 3D Scaffolds with Antibiofilm Properties for Wound Dressings: Optimization of the Microstructure/Nanostructure. ACS Biomater. Sci. Eng..

[267] Brigham C.J., Sinskey A.J. (2012). Application of polyhydroxyalkanoates in the Medical Industry. Int. J. Biotechnol. Wellness Ind..

[268] Shishatskaya E.I., Volova T.G., Puzyr A.P., Mogilnaya O.A., Efremov S.N. (2004). Tissue response to the implantation of biodegradable polyhydroxyalkanoate sutures. J. Mater. Sci. Mater. M.

[269] Elmowafy E., Abdal-Hay A., Skouras A., Tiboni M., Casettari L., Guarino V. (2019). Polyhydroxyalkanoate (PHA): Applications in drug delivery and tissue engineering. Expert Rev. Med. Devices.

[270] Bassas-Galià M., Gonzalez A., Micaux F., Gaillard V., Piantini U., Schintke S., Zinn M., Mathieu M. (2015). Chemical modification of polyhydroxyalkanoates (PHAs) for the preparation of hybrid biomaterials. Chim. Int. J. Chem..

[271] Sadiku E.R., Fasiku V.O., Owonubi S.J., Mukwevho E., Aderibigbe B.A., Lemmer Y., Abbavaram B.R., Manjula B., Nkuna C., Dludlu M.K., Williams H., Kelly P. (2018). Polyhydroxyalkanoates (PHAs) as scaffolds for tissue engineering. Polyhydroxyalkanoates: Biosynthesis, Chemical Structure and Applications.

[272] Braunegg G., Lefebvre G., Genser K.F. (1998). Polyhydroxyalkanoates, biopolyesters from renewable resources: Physiological and engineering aspects. J. Biotechnol..

[273] Rai R., Roether J.A., Knowles J.C., Mordan N., Salih V., Locke I.C., Gordge M.P., McCormick A., Mohn D., Stark W.J. (2017). Highly elastomeric poly(3-hydroxyoctanoate) based natural polymer composite for enhanced keratinocyte regeneration. Int. J. Polym. Mater. Polym. Biomater..

[274] Shishatskaya E.I., Nikolaeva E.D., Vinogradova O.N., Volova T.G. (2016). Experimental wound dressings of degradable PHA for skin defect repair. J. Mater. Sci. Mater. Med..

[275] De Souza L., Shivakumar S., Kalia V. (2019). Polyhydroxyalkanoates (PHA)—Applications in Wound Treatment and as Precursors for Oral Drugs. Biotechnological Applications of Polyhydroxyalkanoates.

[276] Asran A.S., Razghandi K., Aggarwal N., Michler G.H., Groth T. (2010). Nanofibers from Blends of Polyvinyl Alcohol and Polyhydroxy Butyrate As Potential Scaffold Material for Tissue Engineering of Skin. Biomacromolecules.

[277] Gumel A.M., Razaif-Mazinah M.R.M., Anis S.N.S., Annuar M.S.M. (2015). Poly (3-hydroxyalkanoates)-co-(6-hydroxyhexanoate) hydrogel promotes angiogenesis and collagen deposition during cutaneous wound healing in rats. Biomed. Mater..

[278] Li X.-T., Zhang Y., Chen G.-Q. (2008). Nanofibrous polyhydroxyalkanoate matrices as cell growth supporting materials. Biomaterials.

[279] Lim J., You M.L., Li J., Li Z.B. (2017). Emerging bone tissue engineering via Polyhydroxyalkanoate (PHA)-based scaffolds. Mater. Sci. Eng. C Mater. Biol. Appl..

[280] Galego N., Rozsa C., Sánchez R., Fung J., Analía V., Santo-Tomás J. (2000). Characterization and application of poly(β-hydroxyalkanoates) family as composite biomaterials. Polym. Test..

[281] Zhao K., Deng Y., Chen J.C., Chen G.Q. (2003). Polyhydroxyalkanoate (PHA) scaffolds with good mechanical properties and biocompatibility. Biomaterials.

[282] Bretcanu O., Chen Q., Misra S.K., Boccaccini A.R., Roy I., Verne E., Brovarone C.V. (2007). Biodegradable polymer coated 45S5 Bioglassderived glass-ceramic scaffolds for bone tissue engineering. Glass Technol. Eur. J. Glass Sci. Technol. Part A.

[283] Francis L., Meng D., Knowles J.C., Roy I., Boccaccini A.R. (2010). Multi-functional P (3HB) microsphere/45S5 Bioglass^®^-based composite scaffolds for bone tissue engineering. Acta Biomater..

[284] Mouriño V., Cattalini J.P., Roether J.A., Dubey P., Roy I., Boccaccini A.R. (2013). Composite polymer-bioceramic scaffolds with drug delivery capability for bone tissue engineering. Expert Opin. Drug Deliv..

[285] Sodian R., Sperling J.S., Martin D.P., Egozy A., Stock U., Mayer J.E., Vacanti J.P. (2000). Fabrication of a trileaflet heart valve scaffold from a polyhydroxyalkanoate biopolyester for use in tissue engineering. Tissue Eng..

[286] Cheng S.T., Chen Z.F., Chen G.Q. (2008). The expression of cross-linked elastin by rabbit blood vessel smooth muscle cells cultured in polyhydroxyalkanoate scaffolds. Biomaterials.

[287] Rathbone S., Furrer P., Lubben J., Zinn M., Cartmell S. (2010). Biocompatibility of polyhydroxyalkanoate as a potential material for ligament and tendon scaffold material. J. Biomed. Mater. Res. Part. A.

[288] Lizarraga-Valderrama L.R., Taylor C.S., Aeyssens F.C., Haycock J.W., Knowles J.C., Roy I. (2019). Unidirectional neuronal cell growth and differentiation on aligned polyhydroxyalkanoate blend microfibres with varying diameters. J. Tissue Eng. Regen Med..

[289] Bagdadi A.V., Safari M., Dubey P., Basnett P., Sofokleous P., Humphrey E., Locke I., Edirisinghe M., Terracciano C., Boccaccini A.R. (2018). Poly(3-hydroxyoctanoate), a promising new material for cardiac tissue engineering. J. Tissue Eng. Regen Med..

[290] Lizarraga-Valderrama L.R., Nigmatullin R., Taylor C., Haycock J.W., Claeyssens F., Knowles J.C., Roy I. (2015). Nerve tissue engineering using blends of poly(3-hydroxyalkanoates) for peripheral nerve regeneration. Eng. Life Sci..

[291] Francis L., Meng D., Locke I.C., Knowles J.C., Mordan N., Salih V., Boccaccini A.R., Roy I. (2016). Novel poly(3-hydroxybutyrate) composite films containing bioactive glass nanoparticles for wound healing applications. Polym. Int..

[292] Gao S., Tang G., Hua D., Xiong R., Han J., Jiang S., Zhang Q., Huang C. (2019). Stimuli-responsive bio-based polymeric systems and their applications. J. Mat. Chem. B.

[293] Nigmatullin R., Thomas P., Lukasiewicz B., Puthussery H., Roy I. (2015). Polyhydroxyalkanoates, a family of natural polymers, and their applications in drug delivery. J. Chem. Technol. Biotechnol..

[294] Di Mascolo D., Basnett P., Palange A.L., Francardi M., Roy I., Decuzzi P. (2016). Tuning core hydrophobicity of spherical polymeric nanoconstructs for docetaxel delivery. Polym. Int..

[295] Shishatskaya E., Goreva A., Voinova O., Inzhevatkin E., Khlebopros R., Volova T. (2008). Evaluation of antitumor activity of rubomycin deposited in absorbable polymeric microparticles. Bull. Exp. Biol. Med..

[296] Masood F., Chen P., Yasin T., Fatima N., Hasan F., Hameed A. (2013). Encapsulation of Ellipticine in poly-(3-hydroxybutyrate-co-3-hydroxyvalerate) based nanoparticles and its in vitro application. Mater. Sci. Eng. C Mater. Biol. Appl..

[297] Loh X.J., Ong S.J., Tung Y.T., Choo H.T. (2013). Dual responsive micelles based on poly (R)-3-hydroxybutyrate and poly(2-(di-methylamino)ethyl methacrylate) for effective doxorubicin delivery. Polym. Chem..

[298] Xiao L., Wang B., Yang G., Gauthier M. (2012). Poly(Lactic Acid)-Based Biomaterials: Synthesis, Modification and Applications. Biomed. Sci. Eng. Technol..

[299] Msuya N., Katima J.H., Masanja E., Temu A.K. (2017). Poly (lactic acid) Production from Monomer to Polymer: A Review. Scifed J. Polym. Sci..

[300] Inkinen S., Hakkarainen M., Albertsson A.C., Sodergard A. (2011). From lactic acid to poly(lactic acid) (PLA): Characterization and analysis of PLA and its precursors. Biomacromolecules.

[301] Singhvi M.S., Zinjarde S.S., Gokhale D.V. (2019). Polylactic acid: Synthesis and biomedical applications. J. Appl. Microbiol..

[302] Jung Y.K., Kim T.Y., Park S.J., Lee S.Y. (2010). Metabolic engineering of Escherichia coli for the production of polylactic acid and its copolymers. Biotechnol. Bioeng..

[303] Riaz S., Fatima N., Rasheed A., Riaz M., Anwar F., Khatoon Y. (2018). Metabolic Engineered Biocatalyst: A Solution for PLA Based Problems. Int. J. Biomater..

[304] Jung Y.K., Lee S.Y. (2011). Efficient production of polylactic acid and its copolymers by metabolically engineered Escherichia coli. J. Biotechnol..

[305] Elsawy M.A., Kim K.H., Park J.W., Deep A. (2017). Hydrolytic degradation of polylactic acid (PLA) and its composites. Renew. Sustain. Energy Rev..

[306] Pina S., Ferreira J.M.F. (2012). Bioresorbable Plates and Screws for Clinical Applications: A Review. J. Healthc. Eng..

[307] Liu S., Qin S., He M., Zhou D., Qin Q., Wang H. (2020). Current applications of poly(lactic acid) composites in tissue engineering and drug delivery. Compos. Part B Eng..

[308] Prokop A., Jubel A., Helling H.J., Eibach T., Peters C., Baldus S.E., Rehm K.E. (2004). Soft tissue reactions of different biodegradable polylactide implants. Biomaterials.

[309] Majola A., Vainionpaa S., Vihtonen K., Mero M., Vasenius J., Tormala P., Rokkanen P. (1991). Absorption, biocompatibility, and fixation properties of polylactic acid in bone tissue: An experimental study in rats. Clin. Orthop. Relat. Res..

[310] Shikinami Y., Matsusue Y., Nakamura T. (2005). The complete process of bioresorption and bone replacement using devices made of forged composites of raw hydroxyapatite particles/poly l-lactide (F-u-HA/PLLA). Biomaterials.

[311] Hochuli-Vieira E., Cabrini-Gabrielli M.A., Pereira-Filho V.A., Gabrielli M.F., Padilha J.G. (2005). Rigid internal fixation with titanium versus bioresorbable miniplates in the repair of mandibular fractures in rabbits. Int. J. Oral Maxillofac. Surg..

[312] Lassalle V., Ferreira M.L. (2007). PLA Nano- and Microparticles for Drug Delivery: An Overview of the Methods of Preparation. Macromol. Biosci..

[313] Zeng X., Tao W., Liu G., Mei L. (2017). Polydopamine-based surface modification of copolymeric nanoparticles as a targeted drug delivery system for cancer therapy. J. Control. Release.

[314] Mi F.-L., Shyu S.-S., Lin Y.-M., Wu Y.-B., Peng C.-K., Tsai Y.-H. (2003). Chitin/PLGA blend microspheres as a biodegradable drug delivery system: A new delivery system for protein. Biomaterials.

[315] Hu K., Li J., Shen Y., Lu W., Gao X., Zhang Q., Jiang X. (2009). Lactoferrin-conjugated PEG–PLA nanoparticles with improved brain delivery: In vitro and in vivo evaluations. J. Control. Release.

[316] Xia H., Gao X., Gu G., Liu Z., Hu Q., Tu Y., Song Q., Yao L., Pang Z., Jiang X. (2012). Penetratin-functionalized PEG–PLA nanoparticles for brain drug delivery. Int. J. Pharm..

[317] Chuaponpat N., Ueda T., Ishigami A., Kurose T., Ito H. (2020). Morphology, Thermal and Mechanical Properties of Co-Continuous Porous Structure of PLA/PVA Blends by Phase Separation. Polymers.

[318] Buzarovska A., Dinescu S., Chitoiu L., Costache M. (2018). Porous poly (L-lactic acid) nanocomposite scaffolds with functionalized TiO 2 nanoparticles: Properties, cytocompatibility and drug release capability. J. Mater. Sci..

[319] Drechsel E. (1889). Anleitung zur Darstellung Physiologisch Chemischer Präparate.

[320] Shima S., Sakai H. (1977). Polylysine produced by Streptomyces. Agric. Biol. Chem..

[321] Singh M., Rao D.M., Pande S., Battu S., Dutt K.R., Ramesh M. (2011). Medicinal uses of L-lysine: Past and future. Int. J. Res. Pharm. Sci..

[322] Rubia L.B., Gomez R. (1977). TLC sensitivity of six modifications of Dragendorff’s reagent. J. Pharm. Sci..

[323] Wang C., Ren X., Yu C., Wang J., Wang L., Zhuge X., Liu X. (2020). Physiological and Transcriptional Responses of Streptomyces albulus to Acid Stress in the Biosynthesis of ε-Poly-L-lysine. Front. Microbiol..

[324] Hancock R.E. (1997). Peptide antibiotics. Lancet.

[325] Bradshaw J.P. (2003). Cationic antimicrobial peptides. BioDrugs.

[326] Hyldgaard M., Mygind T., Vad B.S., Stenvang M., Otzen D.E., Meyer R.L. (2014). The antimicrobial mechanism of action of epsilon-poly-l-lysine. Appl. Environ. Microb..

[327] Xu M., Song Q., Gao L., Liu H., Feng W., Huo J., Jin H., Huang L., Chai J., Pei Y. (2020). Single-step fabrication of catechol-ε-poly-L-lysine antimicrobial paint that prevents superbug infection and promotes osteoconductivity of titanium implants. Chem. Eng. J..

[328] Wang R., Li Q., Chi B., Wang X., Xu Z., Xu Z., Chen S., Xu H. (2017). Enzyme-induced dual-network ε-poly-L-lysine-based hydrogels with robust self-healing and antibacterial performance. Chem. Commun..

[329] Zou Y.-J., He S.-S., Du J.-Z. (2018). ε-Poly (L-lysine)-based Hydrogels with Fast-acting and Prolonged Antibacterial Activities. Chin. J. Polym. Sci..

[330] Yang X., Wang B., Sha D., Liu Y., Xu J., Shi K., Yu C., Ji X. (2020). Injectable and antibacterial ε-poly (l-lysine)-modified poly (vinyl alcohol)/chitosan/AgNPs hydrogels as wound healing dressings. Polymer.

[331] Karimi M., Yazdi F.T., Mortazavi S.A., Shahabi-Ghahfarrokhi I., Chamani J. (2020). Development of active antimicrobial poly (l-glutamic) acid-poly (l-lysine) packaging material to protect probiotic bacterium. Polym. Test..

[332] Rapp M.V., Maier G.P., Dobbs H.A., Higdon N.J., Waite J.H., Butler A., Israelachvili J.N. (2016). Defining the catechol–cation synergy for enhanced wet adhesion to mineral surfaces. J. Am. Chem. Soc..

[333] Wang R., Li J., Chen W., Xu T., Yun S., Xu Z., Xu Z., Sato T., Chi B., Xu H. (2017). A biomimetic mussel-inspired ε-poly-l-lysine hydrogel with robust tissue-anchor and anti-infection capacity. Adv. Funct. Mater..

[334] Li S., Chen N., Li Y., Li X., Zhan Q., Ban J., Zhao J., Hou X., Yuan X. (2020). Metal-crosslinked ɛ-poly-L-lysine tissue adhesives with high adhesive performance: Inspiration from mussel adhesive environment. Int. J. Biol. Macromol..

[335] Liu S., Liu X., Ren Y., Wang P.H., Pu Y., Yang R., Wang X., Tan X.Y., Ye Z., Maurizot V. (2020). Mussel-inspired Dual-crosslinking Hyaluronic Acid/ε-polylysine Hydrogel with Self-healing and Antibacterial Properties for Wound Healing. ACS Appl. Mater. Interfaces.

[336] De Smedt S.C., Demeester J., Hennink W.E. (2000). Cationic polymer based gene delivery systems. Pharm. Res..

[337] Deng J., Gao N., Wang Y., Yi H., Fang S., Ma Y., Cai L. (2012). Self-assembled cationic micelles based on PEG-PLL-PLLeu hybrid polypeptides as highly effective gene vectors. Biomacromolecules.

[338] Sun Z., Song C., Wang C., Hu Y., Wu J. (2019). Hydrogel-based controlled drug delivery for cancer treatment: A review. Mol. Pharm..

[339] Guo Z., Sui J., Ma M., Hu J., Sun Y., Yang L., Fan Y., Zhang X. (2020). pH-Responsive charge switchable PEGylated ε-poly-l-lysine polymeric nanoparticles-assisted combination therapy for improving breast cancer treatment. J. Control. Release.

[340] El Assal R., Abou-Elkacem L., Tocchio A., Pasley S., Matosevic S., Kaplan D.L., Zylberberg C., Demirci U. (2019). Bioinspired preservation of natural killer cells for cancer immunotherapy. Adv. Sci..

[341] Tarantino L.M. Agency Response Letter GRAS Notice No. GRN 000135. https://www.researchgate.net/publication/237593613_Antimicrobial_Activity_of_e-Polylysine_in_Various_Food_Extracts.

[342] Hiraki J., Ichikawa T., Ninomiya S.-I., Seki H., Uohama K., Seki H., Kimura S., Yanagimoto Y., Barnett J.W. (2003). Use of ADME studies to confirm the safety of ε-polylysine as a preservative in food. Regul. Toxicol. Pharmacol..

[343] Hiraki J., Suzuki E. (1999). Process for Producing ε-poly-L-lysine with Immobilized Streptomyces Albulus. U.S. Patent.

[344] Zhang Y.-X., Perry K., Vinci V.A., Powell K., Stemmer W.P., del Cardayré S.B. (2002). Genome shuffling leads to rapid phenotypic improvement in bacteria. Nature.

[345] Li S., Li F., Chen X.-S., Wang L., Xu J., Tang L., Mao Z.-G. (2012). Genome shuffling enhanced ε-poly-l-lysine production by improving glucose tolerance of Streptomyces graminearus. Appl. Biochem. Biotech..

[346] Li S., Ji J., Hu S., Chen G. (2020). Enhancement of ε-poly-L-lysine production in Streptomyces griseofuscus by addition of exogenous astaxanthin. Bioproc. Biosyst. Eng..

[347] Yamanaka K., Maruyama C., Takagi H., Hamano Y. (2008). ε-Poly-L-lysine dispersity is controlled by a highly unusual nonribosomal peptide synthetase. Nat. Chem. Biol..

[348] Samadlouie H.R., Gharanjik S., Tabatabaie Z.B. (2020). Optimization of the Production of ε-Poly-L-Lysine by Novel Producer Lactic Acid Bacteria Isolated from Traditional Dairy Products. Biomed. Res. Int.

[349] Kimura K., Fujimoto Z. (2010). Enzymatic degradation of poly-gamma-glutamic acid. Amino-Acid Homopolymers Occurring in Nature.

[350] Moghaddam B. (2002). Stress activation of glutamate neurotransmission in the prefrontal cortex: Implications for dopamine-associated psychiatric disorders. Biol. Psychiatry.

[351] Peng L., Hertz L., Huang R., Sonnewald U., Petersen S.B., Westergaard N., Larsson O., Schousboe A. (1993). Utilization of glutamine and of TCA cycle constituents as precursors for transmitter glutamate and GABA. Dev. Neurosci..

[352] Shih L., Van Y.-T. (2001). The production of poly-(γ-glutamic acid) from microorganisms and its various applications. Bioresour. Technol..

[353] Kawashima S., Kanehisa M. (2000). AAindex: Amino acid index database. Nucleic Acids Res..

[354] Tanaka T., Yaguchi T., Hiruta O., Futamura T., Uotani K., Satoh A., Taniguchi M., Susumu O. (1993). Screening for microorganisms having poly (γ-glutamic acid) endohydrolase activity and the enzyme production by Myrothecium sp. TM-4222. Biosci. Biotechnol. Biochem..

[355] Liao Z.-X., Peng S.-F., Ho Y.-C., Mi F.-L., Maiti B., Sung H.-W. (2012). Mechanistic study of transfection of chitosan/DNA complexes coated by anionic poly (γ-glutamic acid). Biomaterials.

[356] Su C.-Y., Tseng C.-L., Wu S.-H., Shih B.-W., Chen Y.-Z., Fang H.-W. (2019). Poly-gamma-glutamic acid functions as an effective lubricant with antimicrobial activity in multipurpose contact lens care solutions. Polymers.

[357] Su C.-Y., Chen C.-C., Chen H.-Y., Lin C.-P., Lin F.-H., Fang H.-W. (2019). Characteristics of an alternative antibacterial biomaterial for mouthwash in the absence of alcohol. J. Dent. Sci..

[358] Sun L., Song L., Zhang X., Zhou R., Yin J., Luan S. (2020). Poly (γ-glutamic acid)-based electrospun nanofibrous mats with photodynamic therapy for effectively combating wound infection. Mater. Sci. Eng. C.

[359] Bae S.-R., Park C., Choi J.-C., Poo H., Kim C.-J., Sung M.-H. (2010). Effects of ultra high molecular weight poly-gamma-glutamic acid from Bacillus subtilis (chungkookjang) on corneal wound healing. J. Microbiol. Biotechnol..

[360] Choi J.-C., Uyama H., Lee C.-H., Sung M.-H. (2015). Promotion effects of ultra-high molecular weight poly-gamma-glutamic acid on wound healing. J. Microbiol. Biotechnol..

[361] Dissemond J., Goos M., Wagner S. (2002). The role of oxidative stress in the pathogenesis and therapy of chronic wounds. Hautarzt Z. Dermatol. Venerol. Verwandte Geb..

[362] Park S.-J., Uyama H., Kwak M.-S., Sung M.-H. (2019). Comparison of the Stability of Poly-γ-Glutamate Hydrogels Prepared by UV and γ-Ray Irradiation. J. Microbiol. Biotechnol..

[363] Hua J., Li Z., Xia W., Yang N., Gong J., Zhang J., Qiao C. (2016). Preparation and properties of EDC/NHS mediated crosslinking poly (gamma-glutamic acid)/epsilon-polylysine hydrogels. Mater. Sci. Eng. C.

[364] Zhang L., Ma Y., Pan X., Chen S., Zhuang H., Wang S. (2018). A composite hydrogel of chitosan/heparin/poly (γ-glutamic acid) loaded with superoxide dismutase for wound healing. Carbohydr. Polym..

[365] Stevanović M., Bračko I., Milenković M., Filipović N., Nunić J., Filipič M., Uskoković D.P. (2014). Multifunctional PLGA particles containing poly (l-glutamic acid)-capped silver nanoparticles and ascorbic acid with simultaneous antioxidative and prolonged antimicrobial activity. Acta Biomater..

[366] Pisani S., Dorati R., Scocozza F., Mariotti C., Chiesa E., Bruni G., Genta I., Auricchio F., Conti M., Conti B. (2020). Preliminary investigation on a new natural based poly (gamma-glutamic acid)/Chitosan bioink. J. Biomed. Mater. Res. Part B Appl. Biomater..

[367] Upadhyay K.K., Bhatt A.N., Mishra A.K., Dwarakanath B.S., Jain S., Schatz C., le Meins J.-F., Farooque A., Chandraiah G., Jain A.K. (2010). The intracellular drug delivery and anti tumor activity of doxorubicin loaded poly (γ-benzyl l-glutamate)-b-hyaluronan polymersomes. Biomaterials.

[368] Liao Z.-X., Peng S.-F., Chiu Y.-L., Hsiao C.-W., Liu H.-Y., Lim W.-H., Lu H.-M., Sung H.-W. (2014). Enhancement of efficiency of chitosan-based complexes for gene transfection with poly (γ-glutamic acid) by augmenting their cellular uptake and intracellular unpackage. J. Control. Release.

[369] Ivanovics G., Erdos L. (1937). Ein beitrag zum wesen der kapselsubstanz des milzbrandbazillus. Z. Immun..

[370] Jang J., Cho M., Chun J.-H., Cho M.-H., Park J., Oh H.-B., Yoo C.-K., Rhie G.-E. (2011). The poly-γ-D-glutamic acid capsule of Bacillus anthracis enhances lethal toxin activity. Infect. Immun..

[371] Candela T., Fouet A. (2006). Poly-gamma-glutamate in bacteria. Mol. Microbiol..

[372] Shurtleff W., Aoyagi A. (2012). History of Natto and Its Relatives (1405–2012).

[373] Luo Z., Guo Y., Liu J., Qiu H., Zhao M., Zou W., Li S. (2016). Microbial synthesis of poly-γ-glutamic acid: Current progress, challenges, and future perspectives. Biotechnol. Biofuels.

[374] Steinkraus K. (2004). Industrialization of Indigenous Fermented Foods, Revised and Expanded.

[375] Feng J., Gu Y., Quan Y., Cao M., Gao W., Zhang W., Wang S., Yang C., Song C. (2015). Improved poly-γ-glutamic acid production in Bacillus amyloliquefaciens by modular pathway engineering. Metab. Eng..

[376] Cai D., He P., Lu X., Zhu C., Zhu J., Zhan Y., Wang Q., Wen Z., Chen S. (2017). A novel approach to improve poly-γ-glutamic acid production by NADPH regeneration in Bacillus licheniformis WX-02. Sci. Rep..

[377] Niemeyer R., Schröder H.C., Müller W.E.G. (1999). Cyclic Condensed Metaphosphates in Plants and the Possible Correlations between Inorganic Polyphosphates and Other Compounds. Inorganic Polyphosphates: Biochemistry, Biology, Biotechnology.

[378] Achbergerova L., Nahalka J. (2011). Polyphosphate—An ancient energy source and active metabolic regulator. Microb. Cell Fact..

[379] Ahn K.H., Kornberg A. (1990). Polyphosphate Kinase from Escherichia-Coli—Purification and Demonstration of a Phosphoenzyme Intermediate. J. Biol. Chem..

[380] Kornberg A. (1995). Inorganic Polyphosphate—Toward Making a Forgotten Polymer Unforgettable. J. Bacteriol..

[381] Tewari K.K., Singh M. (1964). Acid Soluble and Acid Insoluble Inorganic Polyphosphates in Cuscuta-Reflexa. Phytochemistry.

[382] Christ J.J., Blank L.M. (2018). Analytical polyphosphate extraction from Saccharomyces cerevisiae. Anal. Biochem..

[383] Kulaev I.S. (1975). Biochemistry of Inorganic Polyphosphates. Rev. Physiol. Biochem. Pharmacol..

[384] Mandala V.S., Loh D.M., Shepard S.M., Geeson M.B., Sergeyev I.V., Nocera D.G., Cummins C.C., Hong M. (2020). Bacterial Phosphate Granules Contain Cyclic Polyphosphates: Evidence from 31P Solid-State NMR. J. Am. Chem. Soc..

[385] Chaubal M.V., Sen-Gupta A., Lopina S.T., Bruley D.F. (2003). Polyphosphates and other phosphorus-containing polymers for drug delivery applications. Crit. Rev. Drug.

[386] Liu J.Y., Huang W., Pang Y., Yan D.Y. (2015). Hyperbranched polyphosphates: Synthesis, functionalization and biomedical applications. Chem. Soc. Rev..

[387] Liu J.Y., Huang W., Pang Y., Zhu X.Y., Zhou Y.F., Yan D.Y. (2010). Hyperbranched Polyphosphates for Drug Delivery Application: Design, Synthesis, and In Vitro Evaluation. Biomacromolecules.

[388] Vasiliadis G., Duncan A., Bayly R.C., May J.W. (1990). Polyphosphate Production by Strains of Acinetobacter. FEMS Microbiol. Lett..

[389] Liang M., Frank S., Luensdorf H., Warren M.J., Prentice M.B. (2017). Bacterial microcompartment-directed polyphosphate kinase promotes stable polyphosphate accumulation in E. coli. Biotechnol. J..

[390] Zhang H.Y., Ishige K., Kornberg A. (2002). A polyphosphate kinase (PPK2) widely conserved in bacteria. Proc. Natl. Acad. Sci. USA.

[391] Kuroda A., Kornberg A. (1997). Polyphosphate kinase as a nucleoside diphosphate kinase in Escherichia coli and Pseudomonas aeruginosa. Proc. Natl. Acad. Sci. USA.

[392] Xie L.H., Jakob U. (2019). Inorganic polyphosphate, a multifunctional polyanionic protein scaffold. J. Biol. Chem..

[393] Qiu G.L., Zuniga-Montanez R., Law Y.Y., Thi S.S., Nguyen T.Q.N., Eganathan K., Liu X.H., Nielsen P.H., Williams R.B.H., Wuertz S. (2019). Polyphosphate-accumulating organisms in full-scale tropical wastewater treatment plants use diverse carbon sources. Water Res..

[394] Wang X., Wang X.M., Hui K.M., Wei W., Zhang W., Miao A.J., Xiao L., Yang L.Y. (2018). Highly Effective Polyphosphate Synthesis, Phosphate Removal, and Concentration Using Engineered Environmental Bacteria Based on a Simple Solo Medium-Copy Plasmid Strategy. Environ. Sci. Technol..

[395] Wang X.H., Schroder H.C., Muller W.E.G. (2016). Polyphosphate as a metabolic fuel in Metazoa: A foundational breakthrough invention for biomedical applications. Biotechnol. J..

[396] Kulakovskaya T.V., Vagabov V.M., Kulaev I.S. (2012). Inorganic polyphosphate in industry, agriculture and medicine: Modern state and outlook. Process. Biochem..

[397] Maier S.K., Scherer S., Loessner M.J. (1999). Long-chain polyphosphate causes cell lysis and inhibits Bacillus cereus septum formation, which is dependent on divalent cations. Appl. Environ. Microb..

[398] Jen C.M.C., Shelef L.A. (1986). Factors Affecting Sensitivity of Staphylococcus-Aureus 196e to Polyphosphates. Appl. Environ. Microb..

[399] Mutch N.J. (2019). Regulation of Coagulation by Polyphosphate. Blood.

[400] Smith S.A., Choi S.H., Davis-Harrison R., Huyck J., Boettcher J., Reinstra C.M., Morrissey J.H. (2010). Polyphosphate exerts differential effects on blood clotting, depending on polymer size. Blood.

[401] Travers R.J., Smith S.A., Morrissey J.H. (2015). Polyphosphate, platelets, and coagulation. Int. J. Lab. Hematol..

[402] Verhoef J.J.F., Barendrecht A.D., Nickel K.F., Dijkxhoorn K., Kenne E., Labberton L., McCarty O.J.T., Schiffelers R., Heijnen H.F., Hendrickx A.P. (2017). Polyphosphate nanoparticles on the platelet surface trigger contact system activation. Blood.

[403] Wang Y., Li M., Li P., Teng H.J., Fan D.H., Du W.N., Guo Z.L. (2019). Progress and Applications of Polyphosphate in Bone and Cartilage Regeneration. Biomed. Res. Int..

[404] Kawazoe Y., Shiba T., Nakamura R., Mizuno A., Tsutsumi K., Uematsu T., Yamaoka M., Shindoh M., Kohgo T. (2004). Induction of calcification in MC3T3-E1 cells by inorganic polyphosphate. J. Dent. Res..

[405] Leyhausen G., Lorenz B., Zhu H., Geurtsen W., Bohnensack R., Muller W.E.G., Schroder H.C. (1998). Inorganic polyphosphate in human osteoblast-like cells. J. Bone Min. Res..

[406] Schroder H.C., Kurz L., Muller W.E.G., Lorenz B. (2000). Polyphosphate in bone. Biochem. Mosc..

